# Supplementary material for: Fertility and contraceptive dynamics amidst COVID-19: who is at greatest risk for unintended pregnancy among a cohort of adolescents and young adults in Nairobi, Kenya?
Source: BMJ Open. 2023 May 2;13(5):e068689. doi: 10.1136/bmjopen-2022-068689 (PMC10163330; doi:10.1136/bmjopen-2022-068689)
Supplement: Author's manuscript [file bmjopen-2022-068689.draft_revisions.pdf]

BMJ Open is committed to open peer review. As part of this commitment we make the peer review history of every article we publish publicly available.

When an article is published we post the peer reviewers' comments and the authors' responses online. We also post the versions of the paper that were used during peer review. These are the versions that the peer review comments apply to.

The versions of the paper that follow are the versions that were submitted during the peer review process. They are not the versions of record or the final published versions. They should not be cited or distributed as the published version of this manuscript.

BMJ Open is an open access journal and the full, final, typeset and author-corrected version of record of the manuscript is available on our site with no access controls, subscription charges or pay-per-view fees (http://bmjopen.bmj.com).

If you have any questions on BMJ Open's open peer review process please email info.bmjopen@bmj.com

BMJ Open

Fertility and Contraceptive Dynamics amidst COVID-19: Who is at Greatest Risk for Unintended Pregnancy among Adolescents and Young Adults in Nairobi, Kenya?

Journal:	*BMJ Open*
Manuscript ID	bmjopen-2022-068689
Article Type:	Original research
Date Submitted by the Author:	28-Sep-2022

Complete List of Authors:	Wood, Shannon; Johns Hopkins University Bloomberg School of Public Health, Department of Population, Family and Reproductive Health<br>Byrne, Meagan E.; Johns Hopkins University Bloomberg School of Public Health, Department of Population, Family and Reproductive Health<br>Thiongo, Mary; International Centre for Reproductive Health Kenya<br>Devoto, Bianca; Johns Hopkins University Bloomberg School of Public Health, Department of Population, Family and Reproductive Health<br>Wamue-Ngare, G; Kenyatta University, Department of Sociology, Gender and Development Studies<br>Decker, Michele; Johns Hopkins University Bloomberg School of Public Health<br>Gichangi, Peter; International Centre for Reproductive Health Kenya

Keywords:	COVID-19, GYNAECOLOGY, OBSTETRICS, PUBLIC HEALTH, REPRODUCTIVE MEDICINE

SCHOLARONE™
Manuscripts

I, the Submitting Author has the right to grant and does grant on behalf of all authors of the Work (as defined in the below author licence), an exclusive licence and/or a non-exclusive licence for contributions from authors who are: i) UK Crown employees; ii) where BMJ has agreed a CC-BY licence shall apply, and/or iii) in accordance with the terms applicable for US Federal Government officers or employees acting as part of their official duties; on a worldwide, perpetual, irrevocable, royalty-free basis to BMJ Publishing Group Ltd ("BMJ") its licensees and where the relevant Journal is co-owned by BMJ to the co-owners of the Journal, to publish the Work in this journal and any other BMJ products and to exploit all rights, as set out in our licence.

The Submitting Author accepts and understands that any supply made under these terms is made by BMJ to the Submitting Author unless you are acting as an employee on behalf of your employer or a postgraduate student of an affiliated institution which is paying any applicable article publishing charge ("APC") for Open Access articles. Where the Submitting Author wishes to make the Work available on an Open Access basis (and intends to pay the relevant APC), the terms of reuse of such Open Access shall be governed by a Creative Commons licence – details of these licences and which Creative Commons licence will apply to this Work are set out in our licence referred to above.

Other than as permitted in any relevant BMJ Author's Self Archiving Policies, I confirm this Work has not been accepted for publication elsewhere, is not being considered for publication elsewhere and does not duplicate material already published. I confirm all authors consent to publication of this Work and authorise the granting of this licence.

For peer review only

Fertility and Contraceptive Dynamics amidst COVID-19: Who is at Greatest Risk for Unintended Pregnancy among Adolescents and Young Adults in Nairobi, Kenya?

Shannon N. Wood, PhD[1,2]
Meagan E. Byrne, MSPH[1,2]
Mary Thiongo, BS[3]
Bianca Devoto, MSPH[1]
Grace Wamue-Ngare, PhD[4,5]
Michele R. Decker, ScD[1,2]
Peter Gichangi, MD[3,6,7]

[1] Department of Population, Family and Reproductive Health, Johns Hopkins Bloomberg School of Public Health, Baltimore, Maryland, USA.
[2] Bill & Melinda Gates Institute for Population and Reproductive Health, Department of Population, Family and Reproductive Health, Johns Hopkins Bloomberg School of Public Health, Baltimore, Maryland, USA.
[3] International Centre for Reproductive Health-Kenya, Nairobi, Kenya.
[4] Women's Economic Empowerment Hub, Kenyatta University, Nairobi, Kenya.
[5] Department of Sociology, Gender and Development Studies, Kenyatta University, Nairobi, Kenya.
[6] Technical University of Mombasa, Mombasa, Kenya.
[7] Department of Public Health and Primary Care, Faculty of Medicine and Health Sciences, Ghent University, Belgium.

Corresponding Author:
Shannon N. Wood
Johns Hopkins Bloomberg School of Public Health
Department of Population, Family and Reproductive Health
615 N. Wolfe Street Room E4009 Baltimore, MD 21205
swood@jhu.edu
+1 410 502 2303

Word Count: 3,615
Tables: 3
Figures: 3
References: 30
Appendices: 0

For peer review only - http://bmjopen.bmj.com/site/about/guidelines.xhtml

Abstract

Objectives: Among youth in Nairobi, we 1) characterized fertility and contraceptive use dynamics by gender; 2) estimated pregnancy prevalence over the pandemic; 3) assessed factors associated with unintended pandemic pregnancy for young women.

Methods: Longitudinal analyses utilize cohort data from Nairobi youth collected at three time points prior to and during the COVID-19 pandemic: June-August 2019 [pre-pandemic], August-October 2020 [12-month follow-up], and April-May 2021 [18-month follow-up]. Sankey diagrams depicted fertility intentions and contraceptive dynamics, by gender. Among young women, current and period pregnancy prevalence were calculated. Unintended pandemic pregnancy (i.e., current or past 6-month pregnancy with intent to delay pregnancy for more than one year at 2020 survey) was assessed at 18-month follow-up. Bivariate and multivariable logistic regression examined risk factors for unintended pandemic pregnancy.

Results: While fertility intentions remained stable, contraceptive dynamics varied by gender—young men both adopted and discontinued coital-dependent methods, whereas young women adopted coital-dependent or short-acting methods at 12-month follow-up (2020). Current pregnancy was highest at 2020 (4.8%) and hovered at 2% at 2019 and 2021. Unintended pandemic pregnancy prevalence was 6.1%, with increased odds for young women recently married (aOR=3.79; 95% CI=1.83-7.86); recent contraceptive use was protective against unintended pandemic pregnancy (aOR=0.23; 95% CI=0.11-0.47).

Conclusion: The COVID-19 pandemic raised concerns for unintended pregnancy, particularly among young women, however, until now, data on this distal outcome had not been available due to time lag. Youth pregnancy was highest at the height of the COVID-19 pandemic (2020) in Nairobi, and subsided to pre-pandemic levels, however, requires further monitoring. New marriages posed considerable risk for unintended pandemic pregnancy. Contraceptive use, including for married young women, remains a crucial preventive strategy to averting unintended pregnancy.

For peer review only - http://bmjopen.bmj.com/site/about/guidelines.xhtml

Key Messages

What is already known on this topic:

- Prior to the COVID-19 pandemic, sub-Saharan Africa had the highest adolescent pregnancy rates globally, which is of concern due to morbidity, mortality, and well-being impacts for adolescent girls and young women (AGYW).
- The onset of the COVID-19 pandemic increased fears of heightened unintended pregnancy for this high-risk group, however, no study has used prospective data to predict unintended pregnancy during the COVID-19 pandemic.

What this study adds:

- While previous studies have alluded to potential increases in unintended pregnancy for AGYW during the COVID-19 pandemic, to our knowledge this is the first study to longitudinally examine unintended pregnancy incurred during the COVID-19 pandemic among AGYW in sub-Saharan Africa; limitations of retrospective unintended pregnancy assessment are well established, making prospective cohort data uniquely valuable to address this topic.
- Specifically, we found that pregnancy for AGYW in Nairobi was highest in 2020 (4.8%) and hovered at approximately 2% in 2019 and 2021; unintended pregnancy followed similar trends.
- Approximately 6% of AGYW reported an unintended pandemic pregnancy, and odds of unintended pandemic pregnancy were approximately four-fold higher for young women who recently married, compared to those who remained unmarried.

How this study might affect research, practice, or policy:

- Fears of heightened unintended pregnancy during the COVID-19 pandemic were warranted for AGYW in Nairobi, Kenya. Counter to initial hypotheses, education and economic insecurity did not pose significant threats to unintended pandemic pregnancy.
- Pressures for newly married women may increase risk of unintended pregnancy, both during the early stages of the COVID-19 pandemic and after, and require further research to understand relationship dynamics for newly married couples.

For peer review only

Introduction

Adolescent pregnancy is a human rights concern with gendered repercussions that disproportionately impact adolescent girls and young women (AGYW) (1,2). Both intended and unintended pregnancies can alter AGYW's life trajectories, including by decreasing their ability to meet educational, business/financial, and family goals (1). Further, early pregnancy can have negative health effects—adolescents are at risk for increased birth complications, including obstructed labor, fistula, premature delivery, low birth weight, pre-eclampsia/eclampsia, and maternal and perinatal mortality, compared to their older counterparts (1,3). While complete adolescent pregnancy data are difficult to obtain, largely due to underreporting and stigmatization of unintended pregnancies that end in abortions, the evidence is clear that the majority of the global adolescent pregnancy burden is concentrated within sub-Saharan Africa, where adolescent pregnancy rates in most countries exceed 100 per 1,000 women aged 15-19 (4).

Motivations and intentions surrounding adolescent pregnancy are complex and require a socioecological lens (5). Pregnancy intendedness varies substantially by setting and in many contexts, cultural and familial norms may promote early childbearing in lieu of economic and educational attainment (2). Factors linked to adolescent pregnancy in sub-Saharan Africa include low education, lack of familial education, poverty, and limited communication/knowledge surrounding sexual and reproductive health (SRH) (6,7). Ecological data further correlate adolescent pregnancy with high gender inequality at the national level (8). While rural adolescents are generally at heightened risk of pregnancy, largely due to decreased access to SRH services (6,7), urban adolescents have long been a target group for SRH programs given the population's growing size and unique needs (9). Many AGYW migrate to cities to seek out financial opportunities, but instead are met with stigma and shame when seeking healthcare services (10,11); these barriers may be more pronounced for unmarried youth given social norms dictating sex as a means for procreation and stigmatization of sexual activity outside of marriage, and in turn, increase unmet SRH needs (12).

When the COVID-19 outbreak was declared a pandemic by the World Health Organization (WHO) in March 2020, the SRH community was concerned about the negative impact mitigation measures would have on women's SRH, and particularly the SRH of AGYW (13–15). Early COVID-19 projections estimated that 15 million additional unintended pregnancies could occur over one year if COVID-related service disruptions affected 10% of women in need of SRH services in low- and middle-income countries (16). These projections, however, have not been evaluated with data given that unintended pregnancy is a distal outcome and requires time and prospective data to accurately assess. Thus far, contraceptive use is the most widely used proxy for unintended pregnancy—population-level data from four sub-Saharan African contexts indicate that contraceptive use among women in need increased across settings, contrary to warnings of unintended pregnancy increases (17). While early trends are largely promising, some groups may be at increased risk of unintended pregnancy, namely adolescents and nulliparous women, due to increases in contraceptive need and reductions in contraceptive use during this period (17). Another study utilizing facility data to assess facility admissions and antenatal, delivery, and immunization services reported slight reductions in services (18). Adolescents may further face difficulties procuring contraception during the COVID-19 pandemic given limited household bargaining power and school closures (19,20). Recent causal-comparative research from Western Kenya elucidates increased school dropout and pregnancy among specific to secondary school girls during COVID-19 compared to a pre-COVID-19 cohort (21).

While multiple studies have examined youth contraceptive use and/or sexual activity in light of COVID-19 (19,20), no study to date has utilized prospective data to predict unintended pregnancy during the COVID-19 pandemic in sub-Saharan Africa. Among urban youth in Nairobi, Kenya, this study aimed to 1) characterize fertility and contraceptive use dynamics by gender; 2) estimate pregnancy prevalence for AGYW over the course of the pandemic; and 3) assess factors associated with unintended pandemic pregnancy for young women.

Methods

Study Setting

Kenya has experienced a gradual decline in total fertility rates, from 4.9 in 2003 to 3.9 in 2014 (22); however, adolescent pregnancy remains a country priority (23). Per most recent Demographic and Health Survey estimates (2014), one-fifth of Kenyan AGYW ages 15-19 were pregnant or already mothers (22). Among unmarried, sexually active AGYW, approximately half reported using a modern method of contraception (22).

The first case of COVID-19 in Kenya was detected on March 13, 2020 (24), with school closures, national lockdown, and mandatory curfew immediately following (25). By late 2020 restrictions were eased; however, in summer 2021,

For peer review only - http://bmjopen.bmj.com/site/about/guidelines.xhtml

caseloads began rising again, prompting additional closures (25). These restrictions, while essential to curbing the spread of COVID-19, could decrease access to essential health services, including SRH services.

Study Overview

This longitudinal study draws on data collected from the Nairobi Youth Respondent Driven Sampling Survey (YRDSS), a cohort of adolescents and young adults in Nairobi collected at three time points prior to and during the COVID-19 pandemic: June-August 2019 [pre-pandemic], August-October 2020 [12-month follow-up], and April-May 2021 [18-month follow-up].

Briefly, the YRDSS began recruitment in June-August 2019 using respondent-driven sampling (RDS), a chain-based recruitment method that begins with purposefully selected seeds, followed by monitored peer-to-peer coupon distribution (26). At the time of recruitment, eligible youth were aged 15-24 years, unmarried, and residing in Nairobi for at least one year. The 2019 pre-pandemic survey round recruited 1,357 participants; of whom 95% (1,293/1,357) consented for recontact and provided contact information. This cohort was recontacted and surveyed in August-October 2020 (12-month follow-up; n=1,217; 94% retention], and in April-May 2021 (18-month follow-up; n=1,177; 97% retention).

Ethical approval was obtained from the Ethics Review Committee at Kenyatta National Hospital/University of Nairobi (P294/04/2019) and the Institutional Review Board at Johns Hopkins Bloomberg School of Public Health (00009496). Detailed study procedures are outlined elsewhere (27).

Data Collection Procedures

The pre-pandemic [2019] survey was specific to reproductive health needs and family planning behaviors for adolescents and young adults. Data collection occurred in-person with surveys self-administered by participants or administered by trained resident enumerators in case of comprehension or technological difficulty. Data was entered onto tablets and uploaded to a cloud server using Open Data Kit (ODK) software. Surveys were conducted in English or Swahili, based on participant preference.

Given the onset of the COVID-19 pandemic, follow-up surveys at 12-month [2020] and 18-month [2021] follow-up were designed specifically to examine the gendered impacts of COVID-19 on youth economic, health, and social experiences, including pandemic impacts on youth contraceptive use and unintended pregnancy. In adherence to COVID-19 restrictions, interviews at these timepoints were conducted by the same resident enumerators via remote phone interviews with responses recorded using the ODK software.

Measures

Outcomes. Primary outcomes comprised fertility and contraceptive use for both genders, as well as pregnancy for young women.

Prospective fertility intentions were assessed per gender at each of the three time points. Specifically, each participant was asked "How long would you like to wait from now before the birth of a child?" with responses comprising "x months", "x years", "would like to get pregnant soon/now", and "can't get pregnant" (for women) or "can't cause pregnancy" (for men), and "do not want children." For trend analysis, intentions were categorized as "currently pregnant"; "<1 year"; "1-2 years"; ">2 years/other."

Current contraceptive use was assessed per gender at each time point utilizing standard items (28). Contraceptive users were asked current method, and if users reported more than one current method, they were then asked to identify the method they used "mainly" or "most of the time". Responses were split into method effectiveness categories for trend analysis (1-long-acting reversible contraception (LARC): intrauterine device (IUD) and implant; 2-short-acting: injectable and pills; 3-coital-dependent: emergency contraception, male condoms, and female condoms; 4-other/traditional: rhythm, withdrawal, and herbal pill method).

Period and current pregnancy were assessed per time point for young women. At pre-pandemic baseline, young women were asked about ever pregnancy, whereas at 12-month and 18-month follow-up, past 12-month and past 6-month pregnancy were assessed to capture between-survey outcomes. For all young women with a period pregnancy, current pregnancy assessed via single item, "Are you currently pregnant?" A dichotomous variable was then created for the primary outcome of interest, unintended pandemic pregnancy, defined as having a current pregnancy or past

For peer review only - http://bmjopen.bmj.com/site/about/guidelines.xhtml

6-month pregnancy at 18-month follow-up survey and wanting to wait more than one year for a pregnancy at last survey round (2020).

<u>Exposures for unintended pandemic pregnancy</u>: All risk factors for unintended pandemic pregnancy were assessed at 12-month follow-up (2020) survey using standard measures (27,28), and include age (15-19; 20-24); highest level of education completed (no school/primary/post-primary; secondary or higher); subjective household wealth tertile (low; middle; high); employment (someone else vs. self); increased time with partner since COVID-19 restrictions (yes/no); ability to meet basic needs (yes/no); and contraceptive use at 2020 survey. Exposures were dichotomized based on underlying distributions to maximize power for multivariable analyses.

Analytic Sample

Within timepoint analyses were restricted to participants with completed survey data for that round (n=605 young men, n=610 young women at pre-pandemic and 12-month follow-up; n=586 young men, n=591 young women at 18-month follow-up). Trend and prospective analyses were restricted to those with complete data at all three timepoints (n=586 young men, n=589 young women); for contraceptive dynamics, analyses were additionally restricted to those with a need for contraception (i.e., sexually active, not pregnant (if female), and not wanting a child within the next year) at both the time of that survey and the prior survey round. A complete case approach was adopted given <1% missing outcome data (n=2 missing unintended pandemic pregnancy); sample size floats to accommodate small amounts of missing covariate data (<1%).

Statistical Analysis

Descriptive statistics examined characteristics of youth at pre-pandemic, by gender, with design-based F-statistics classifying between-gender differences. Sankey diagrams depicted dynamics in fertility intentions and contraceptive use across the three survey rounds, by gender. Among youth who discontinued their method at 12-month or 18-month follow-up, main reason for discontinuation was examined, per gender and time point. For each round, period and current pregnancy prevalence were calculated for all young women, and among adolescent girls (<18-years-old) and those wishing to avert a pregnancy at last round (i.e., did not want a child within the next year). Lastly, bivariate and multivariable logistic regression examined factors at 12-month follow-up (2020) associated with unintended pandemic pregnancy among young women via glm models with family binomial and link logistic to account for weighing and clustering. Factors with p-values <0.1 from bivariate models were included within multivariable models, withstanding increased time with partner, omitted due to collinearity.

All analyses were conducted using Stata 16 (College Station, TX) with statistical significance set a priori at $p<0{\cdot}05$. Sampling weights accommodate the respondent driven sampling study design using RDS-II (Volz-Heckathorn) weights, post-estimation adjustment based on 2014 Kenya Demographic and Health Survey population data (age, sex, education levels), and loss-to-follow-up. All estimates are weighted; statistical testing accounts for clustering among participants recruited by the same recruiter at baseline.

Patient and Public Involvement

This community-engaged study sought public and end-user input at all phases. During the formative research stage prior to the 2019 cohort recruitment, input from community-based, youth-serving organizations informed the study recruitment strategy for feasibility, survey measures and constructs to ensure relevance, and study logistics to maximize participant comfort and confidentiality. All recruitment and procedures were conducted by trained resident enumerators selected from underlying communities, and who provided inputs on measures for clarity and aided in results interpretation. Findings were disseminated in November 2020 and again in September 2021 with stakeholders spanning policy sector, government representatives, elders/faith leaders, community-based organizations, and youth leaders from the study communities.

Results

Characteristics of youth at pre-pandemic, by gender, are outlined in Table 1. The majority of both young men and young women were aged 20-24 (60.7% and 57.2%, respectively) and had completed at least secondary education (62.5% and 57.2%, respectively). While roughly three of four youth had ever had sex, higher proportions of young men reported current involvement in a romantic relationship (young men: 66.5%; young women: 54.5%; p=0.04). Approximately one in ten young men had a partner who had ever been pregnant and 8.0% of young women reported their own pregnancy experience. Future fertility intentions differed by gender, with higher proportions of young men reporting wanting a child soon/now (2.7% young men vs. 0.2% young women) or within the next two years (15.8%

For peer review only - http://bmjopen.bmj.com/site/about/guidelines.xhtml

young men vs. 11.0% young women; p=0.05). Contraceptive use, method effectiveness, and main current contraceptive method differed significantly by gender. Approximately three-quarters of young men reported current contraceptive use (75.2%), compared to half of young women (54.7%; p<0.001). The majority (91.2%) of young men reported male condom use as their main contraceptive method, whereas young women utilized a range of methods (p<0.001).

Table 1. Characteristics of youth at pre-pandemic (2019; n=1,215), weighted

	Young Men (n=605)	Young Women (n=610)	p-value[§]
	n (column %)		
Demographic Characteristics			
Age			0.38
15-19	238 (39.3)	261 (42.8)	
20-24	367 (60.7)	349 (57.2)	
Highest level of education completed			0.62
Post-primary/primary or no school	197 (32.6)	211 (34.7)	
Secondary or higher	408 (67.4)	399 (65.4)	
Relationship and Reproductive Characteristics			
Ever had sex	436 (72.0)	441 (72.3)	0.94
Currently in a romantic/sexual relationship	402 (66.5)	332 (54.5)	**0.04**
Ever pregnant/partner has ever been pregnant	62 (10.2)	49 (8.0)	0.35
Future fertility intentions			**0.05**
Soon/now	16.3 (2.7)	1 (0.2)	
Within the next two years	96 (15.8)	67 (11.0)	
More than two years	410 (67.7)	446 (73.2)	
Does not want children or cannot have children	7 (1.1)	8 (1.4)	
Don't know	76 (12.6)	87 (14.2)	
Contraceptive Characteristics			
Contraceptive use	323 (75.2)	249 (54.7)	**<0.001**
Method effectiveness[±]			**<0.001**
Long-acting reversible contraception	3 (1.0)	48 (19.5)	
Short-acting	2 (0.5)	55 (22.5)	
Barrier or hormonal coital-dependent	286 (93.6)	114 (46.5)	
Other/traditional	15 (4.9)	28 (11.4)	
Main Contraceptive Method[±]			**<0.001**
Implant	3 (1.0)	48 (19.1)	
Coil/IUD	0 (0.0)	7 (2.7)	
Injectable	2 (0.5)	43 (17.0)	
Oral contraceptive pills	0 (0.0)	12 (5.0)	
Emergency contraception	6 (1.9)	24 (9.7)	
Male condom	281 (91.2)	85 (34.0)	
Female condom	0 (0.0)	5 (1.8)	
Cycle beads/Standard Days Method/rhythm	7 (2.4)	12 (4.6)	
Withdrawal	8 (2.5)	10 (3.9)	
Herbal Pill Method	1 (0.5)	5 (2.1)	
Place Obtained Main Contraceptive Method[±]			0.28
Hospital	26 (9.1)	34 (15.1)	
Health Center	89 (30.1)	69 (30.9)	
Clinic/Other Health Provider	26 (9.1)	31 (13.7)	

For peer review only - http://bmjopen.bmj.com/site/about/guidelines.xhtml

Pharmacy	91 (31.7)	62 (27.6)	
Other	55 (19.1)	28 (12.7)	

§p-value from design-based F-statistic; boldface indicates $p<0.05$
±Among users of contraception
Method effectiveness: LARC=IUD, implant; short-acting=injectable, pills; coital-dependent=emergency contraception, male condoms, female condoms; other/traditional=rhythm, withdrawal, herbal pill method
Bolding indicates $p<0.05$

Throughout the pandemic, young women's fertility intentions remained relatively stable (Figure 1). Between pre-pandemic and 12-month follow up [2020], largest shifts in fertility intention were seen among women who wanted to wait more than two years at pre-pandemic (88.6%; n=515/591) and then said they wanted a child in 1-2 years at 2020 (11.4%; n=80/591); this trend was similarly observed between 12-month and 18-month follow-up, wherein 9.9% of all women (n=65/591) shifted from the greater than 2 years category to 1-2 years. The second largest shift was seen among young women who wanted to wait more than two years at pre-pandemic, but then reported pregnancy at 12-month [2020] follow-up (4.2%; n=17/591). From 12-month to 18-month follow-up, a corresponding shift from pregnancy to wanting to wait more than two years was reported (4.8%; n=20/591).

Trends in fertility intentions for young men (Figure 1) were similar; however, between 12-month [2020] and 18-month [2021] follow-up, large portions of young men also sought to delay childbearing, with 6.8% changing from wanting a child within 1-2 years to wanting to wait two or more years (n=35/586).

[Figure 1 here]

Contraceptive dynamics throughout the pandemic showed substantial variation by gender (Figure 2). Specifically, young men largely migrated between coital-dependent method use and non-use at both timepoints (including both adoption and discontinuation). Conversely, many young women began as non-users and adopted coital-dependent (33.5%; n=67/192) or short-acting methods (13.3%; n=30/192) at 12-month follow-up [2020]. While some coital-dependent users at 12-month follow-up [2020] discontinued by 18-month follow-up (26.2%; n=28/113) [2021], some non-users also continued to take up coital-dependent (15.2%; n=25/120), short-acting (5.0%; n=6/120), and LARC methods (11.4%; 12/120).

[Figure 2 here]

At both 2020 and 2021 surveys and across genders, the primary reason for contraceptive discontinuation centered around partners, including fear of cheating, partner opposed, partner away, infrequent sex, not married (Young women_{2020}: 64%; Young women_{2021}: 62%; Young men_{2020}: 66%; Young men_{2021} 76%; Figure 3). Additionally, approximately one-fifth of both young women (21%) and young men (22%) at 2020 survey stated discontinuation for pregnancy-related reasons, namely wanting to become pregnant. COVID-related, access-related, and personal concerns were less common.

[Figure 3 here]

Following trends in fertility intentions and contraceptive use, prevalence of pregnancy differed across survey rounds (Table 2). Period prevalence ranged from 31.4% ever pregnant at pre-pandemic (2019) to 6.8% pregnant in the past 6-months at 18-month follow-up (2021). Current pregnancy was highest at the 12-month follow-up survey (2020) at 4.8%, whereas it hovered at approximately two percent at the 2019 and 2021 survey rounds. Unintended pandemic pregnancy (i.e., current pregnancy or past 6-month pregnancy at the 18-month follow-up survey with no intention to get pregnant indicated at the previous survey round) was 6.1%, with prevalence approximately half for girls less than age 18 (3.3%).

For peer review only - http://bmjopen.bmj.com/site/about/guidelines.xhtml

Table 2. Prevalence of pregnancy across survey rounds, weighted

	All AGYW	Adolescent girls (<18 years old at baseline)	Among AGYW wishing to avert a pregnancy at last round[§]
	% (n)		
Period prevalence			
Pre-pandemic (2019) ever pregnant	31.4 (191)	6.2 (5)	--
12-month follow-up (2020) past 12-month pregnancy	12.5 (76)	1.2 (1)	11.2 (65)
18-month follow-up (2021) past 6-month pregnancy	6.8 (40)	3.3 (1)	6.6 (36)
Current pregnancy			
Pre-pandemic (2019)	1.8 (11)	0.0 (0)	--
12-month follow-up (2020)	4.8 (29)	0.0 (0)	4.6 (27)
18-month follow-up (2021)	2.0 (12)	3.3 (1)	1.6 (9)
Unintended pandemic pregnancy (current pregnancy or past-6-month pregnancy at 18-month follow-up, with no intention to get pregnant at last round (2020)	6.1 (36)	3.3 (1)	--

§Wished to wait for pregnancy >1 year at pre-pandemic (for 12-month follow-up assessment) and >6 months at 12-month follow-up (for 18-month follow-up assessment)

Table 3 presents bivariate and multivariable factors at 12-month follow-up (2020) associated with unintended pandemic pregnancy. Recent marriage (at 2020 survey) was associated with a greater than four-fold increase in unintended pandemic pregnancy in both bivariate (OR=4.89; 95% CI=1.93-12.37; p<0.001) and multivariable models (aOR=4.16; 95% CI=1.65-10.51; p=0.006). Within the bivariate model, increased time with partner since COVID-19 restrictions was also associated with increased risk of unintended pandemic pregnancy (OR=3.69; 95% CI=1.61-8.46; p=0.002), however, was excluded from multivariable models due to collinearity with marital status. Contraceptive use at 2020 survey contributed to an 80% reduction in unintended pandemic pregnancy (aOR=0.21; 95% CI=0.09-0.50; p<0.001). Additionally, within both bivariate models, young women with an inability to meet basic needs displayed a borderline protective effect against unintended pandemic pregnancy (OR=0.47; 95% CI=0.21-1.05; p=0.07); however, this association attenuated when adjusted.

Table 3. Factors at 12-month Follow-up (2020) Associated with Unintended Pandemic Pregnancy for Young Women (n=589)

	Unintended Pandemic Pregnancy Row %	OR (95% CI)	aOR (95% CI)
Factors Assessed at 12-month follow-up (2020)			
Age			
15-19	6.5	Ref	--
20-24	5.9	0.90 (0.37, 2.19)	--
Highest level of education completed			
Post-primary/primary or no school	6.9	Ref	--
Secondary or higher	5.7	0.82 (0.32, 2.08)	--
Household wealth			
Low	6.4	Ref	--
Middle	5.6	0.87 (0.33, 2.34)	--
High	6.2	0.96 (0.34, 2.70)	--
Married			
No	4.5	Ref	ref
Yes	18.9	**4.89 (1.93, 12.37)** ***	**4.16 (1.65, 10.51)** **
Employed			
Student, caregiver, unemployed	5.1	Ref	--
Employed formally or informally	7.2	1.45 (0.60, 3.49)	--
Prime earner in household			

For peer review only - http://bmjopen.bmj.com/site/about/guidelines.xhtml

Someone else	5.9	Ref	--
Self	7.4	1.28 (0.46, 3.57)	--
Increased time with partner since COVID-19 restrictions			
No	3.8	Ref	--
Yes	12.8	**3.69 (1.61, 8.46)** [**]	--
Ability to meet basic needs			
No	8.4	0.47 (0.21, 1.05) [œ]	0.54 (0.23, 1.29) [œ]
Yes	4.1	Ref	ref
Contraceptive use			
No	14.4	Ref	ref
Yes	3.7	**0.23 (0.10, 0.53)** [***]	**0.21 (0.09, 0.50)** [***]

Increased time with partner omitted from multivariable model due to collinearity
Bolding indicates $p<0.05$

Discussion

Within a narrative that has largely focused on shifting fertility intentions and contraceptive use amidst the COVID-19 pandemic (16,17,29), these longitudinal results indicate that fears of unintended pregnancy were indeed warranted. Results on fertility intentions, contraceptive use, and pregnancy all tell the same story—AGYW decreased their contraceptive use and in turn, experienced unintended pregnancy, during the time of highest COVID-19 mitigation measures (2020 survey). Specifically, current pregnancy more than doubled at the 2020 survey wave (4.8%) versus pre-pandemic (1.8%) or 2021 (2.0%) surveys, and the majority of these pregnancies (93%) were unintended. Counter to initial hypotheses, education and economic insecurity did not pose significant threats to unintended pandemic pregnancy—instead, risk among AGYW largely centered on marriage.

Notably, recent marriage was the only risk factor associated with increased odds of unintended pandemic pregnancy within the multivariable models, where young women recently married had a four-fold increase of unintended pandemic pregnancy, as compared to those unmarried ($p<0.001$). Such findings bolster recent results from national cohorts of adult women predicting increases in unintended pregnancy for nulliparous and young women in Lagos, Nigeria (17); notably, however, this research did not highlight potential increases in unintended pregnancy for young women within Kenya. Utilizing longitudinal data from an existing cohort of this hard-to-reach population, our findings elucidate that it may not be age nor parity driving risk for AGYW, but rather power dynamics within a newly formed marriage. Pressures for newly married women may increase risk of unintended pregnancy, both during the COVID-19 pandemic and after, and require further research to understand relationship dynamics for newly married couples. While programs to increase age at marriage and keep girls in school are vital to ensure AGYW's autonomy in the decision to marry and their future livelihoods, interventions to increase relationship quality and help young couples navigate the complexities of this new life stage must be simultaneously prioritized in order for young couples to prosper both individually and jointly.

Shifts in fertility intentions throughout the pandemic largely followed expected patterns and timelines (i.e., women who wanted to wait more than two years at pre-pandemic stated that they then wanted a child within 1-2 years one year later). Some AGYW (4.2%; n=17), however, did report wanting to wait more than two years at pre-pandemic survey but then had an unintended pregnancy at 2020 survey—at 2021 survey, these young women again wanted to wait more than two years for their next pregnancy. While young men's fertility intentions followed similar patterns, between 12-month [2020] and 18-month [2021] follow-up, 6.8% shifted from wanting a child within 1-2 years to wanting to wait two or more years—this shift in intentions could be in part due to the continued economic uncertainty and instability imposed by the COVID-19 pandemic. Results corroborate borderline significant results in multivariable models indicating that AGYW with an inability to meet their basic needs had a 50% reduction in unintended pandemic pregnancy. Ultimately, these results speak to the resilience of young people, as well as their initiative to prepare and financially plan for a pregnancy.

Moreover, results affirm the effectiveness of primary prevention for unintended pregnancy via contraceptive use. Contraceptive use at previous survey round was the only factor protective against unintended pandemic pregnancy—AGYW who used contraception, regardless of method, had an 80% reduction in unintended pandemic pregnancy, compared to those who were not using contraception at last survey round. Youth must have continued access to non-

For peer review only - http://bmjopen.bmj.com/site/about/guidelines.xhtml

stigmatizing, youth-friendly, and low-cost SRH services. For AGYW who experience unintended pregnancies, during the COVID-19 pandemic and otherwise, access to postpartum contraception is imperative to deter against rapid repeat pregnancy, while ensuring that timing of future pregnancies aligns with a woman's reproductive intentions.

Results should be interpreted considering study limitations. Foremost, data were collected immediately prior to the pandemic and at two points after the implementation of COVID-19 restrictions in Nairobi—as Kenya continues to experience COVID-19 infection waves and reinstates mobility restrictions to curb infection spread, ongoing data collection is needed to understand impacts on AGYW's SRH and well-being. Moreover, the assessment of unintended pandemic pregnancy, while our best estimate, may be subject to measurement error given that data collection was not continually ongoing throughout the pandemic. Namely, some AGYW who were pregnant at the 2020 survey round may have indeed experienced an unintended pandemic pregnancy, although we utilized the 18-month (2021) survey for more accurate estimates. Nairobi is a major urban center and results should not be generalized to all parts of Kenya, where AGYW may face different challenges surrounding SRH.

While as of June 2022, Kenya is experiencing a sixth wave of COVID-19 infections with health and economic impacts yet to be fully uncovered, recent notable policy shifts signal positive steps for youth in the country (30). Growing momentum seeks to advance women's safety and reproductive rights, including via affirming access to abortion services, and advocating for the elimination of gender-based violence as a national priority. These prospective data, however, highlight that there is still considerable work to be done to affirm reproductive rights and access to quality SRH care for adolescents and young adults. Evidence-informed policy efforts should center on dismantling fertility pressures within the community and partner dyad, particularly for AGYW transitioning from dating to marital relationships while still seeking to avert pregnancy; continued data collection can help understand the SRH needs of this vulnerable group. To ultimately protect the reproductive autonomy of adolescents and young adults, continuous access to contraception, health care services, and SRH information is imperative during both global crises and life course transitions.

For peer review only

Funding: This work was supported, in whole, by the Bill & Melinda Gates Foundation [010481]. Under the grant conditions of the Foundation, a Creative Commons Attribution 4.0 Generic License has already been assigned to the Author Accepted Manuscript version that might arise from this submission. The funders had no role in the study design, collection, analysis, and interpretation of data, in writing of the report, or in the decision to submit for publication.

Contributors:
Shannon N. Wood: study design, literature search, data analysis, data interpretation, writing
Meagan E. Byrne: study design, literature search, data analysis, figures, data interpretation, writing
Mary Thiongo: study design, data interpretation, writing
Bianca Devoto: study design, data interpretation, writing
Grace Wamue-Ngare: data interpretation, writing
Michele R. Decker: study design, data interpretation, writing
Peter Gichangi: study design, data collection, data interpretation, writing
All authors read and approved the final version of this manuscript.

Ethics Approval: Ethical approval was obtained from the Ethics Review Committee at Kenyatta National Hospital/University of Nairobi (P294/04/2019) and the Institutional Review Board at Johns Hopkins Bloomberg School of Public Health (00009496).

Declaration of interests: No conflicts of interest to disclose.

Acknowledgements: We acknowledge Dr. Caroline Moreau for her assistance in defining our analytic sample and outcome variables.

Data Sharing: Data are available upon request from www.pmadata.org.

For peer review only

References

1. UNFPA. Motherhood in Childhood [Internet]. The State of the World Population 2013. 2013. Available from: http://www.unfpa.org/sites/default/files/pub-pdf/EN-SWOP2013.pdf
2. Chandra-Mouli V, Camacho AV, Michaud PA. WHO guidelines on preventing early pregnancy and poor reproductive outcomes among adolescents in developing countries. J Adolesc Heal. 2013;52(5):517–22.
3. Grønvik T, Sandøy IF. Complications associated with adolescent childbearing in Sub-Saharan Africa: A systematic literature review and meta-analysis. PLoS One. 2018;13(9):1–21.
4. Sedgh G, Finer LB, Bankole A, Eilers MA, Singh S. Adolescent Pregnancy, Birth, and Abortion Rates Across Countries: Levels and Recent Trends. J Adolesc Heal [Internet]. 2015;56(2):223–30. Available from: http://dx.doi.org/10.1016/j.jadohealth.2014.09.007
5. Blum RW, Bastos FI, Kabiru CW, Le LC. Adolescent health in the 21st century. Lancet. 2012;379(9826):1567–8.
6. Kassa GM, Arowojolu AO, Odukogbe AA, Yalew AW. Prevalence and determinants of adolescent pregnancy in Africa: A systematic review and Meta-analysis 11 Medical and Health Sciences 1117 Public Health and Health Services. Reprod Health. 2018;15(1):1–17.
7. Yakubu I, Salisu WJ. Determinants of adolescent pregnancy in sub-Saharan Africa: A systematic review. Reprod Health. 2018;15(1).
8. Decker MR, Kalamar A, Tunçalp Ö, Hindin MJ. Early adolescent childbearing in low- and middle-income countries: Associations with income inequity, human development and gender equality. Health Policy Plan. 2017;32(2):277–82.
9. Melesse DY, Mutua MK, Choudhury A, Wado YD, Faye CM, Neal S, et al. Adolescent sexual and reproductive health in sub-Saharan Africa: Who is left behind? BMJ Glob Heal. 2020;5(e002231).
10. Stidham Hall K, Manu A, Morhe E, Dalton V, Challa S, Loll D, et al. Bad girl and unmet family planning need among Sub-Saharan African adolescents: the role of sexual and reproductive health stigma. Qual Res Med Healthc. 2018;2(1):55–64.
11. Wood K, Jewkes R. Blood Blockages and Scolding Nurses: Barriers to Adolescent Contraceptive Use in South Africa. Reprod Health Matters. 2006;14(27):109–18.
12. Chandra-Mouli V, McCarraher DR, Phillips SJ, Williamson NE, Hainsworth G. Contraception for adolescents in low and middle income countries: Needs, barriers, and access. Reprod Health. 2014;11(1):1–8.
13. Hussein J. COVID-19: What implications for sexual and reproductive health and rights globally? Sex Reprod Heal Matters. 2020;28(1).
14. Thomas AB, Yaya S. Looking ahead in the COVID ‑ 19 pandemic : emerging lessons learned for sexual and reproductive health services in low‑ and middle‑ income countries. Reprod Health. 2021;4:1–8.
15. Marie Stopes International. COVID-19 threatens women's health [Internet]. 2020. Available from: https://mariestopes-us.org/2020/covid-19-threatens-womens-health/
16. Riley T, Sully E, Ahmed Z, Biddlecom A. Estimates of the Potential Impact of the COVID-19 Pandemic on Sexual and Reproductive Health In Low- and Middle-Income Countries. Int Perspect Sex Reprod Health. 2020;46:73–6.
17. Wood SN, Karp C, OlaOlorun F, Akilimali P, Guiella G, Gichangi P, et al. Trends in the need for and use of contraception before and during COVID-19 in four sub-Saharan African geographies: results from population-based national or regional cohort surveys. Lancet Glob Heal. 2021;9:e793-801.
18. Amouzou A, Maïga A, Faye CM, Chakwera S, Melesse DY, Mutua MK, et al. Health service utilisation during the COVID-19 pandemic in sub-Saharan Africa in 2020: A multicountry empirical assessment with a focus on maternal, newborn and child health services. BMJ Glob Heal. 2022;7(5).
19. Karp C, Moreau C, Sheehy G, Anjur-Dietrich S, Mbushi F, Muluve E, et al. Youth Relationships in the Era of COVID-19: A Mixed-Methods Study Among Adolescent Girls and Young Women in Kenya. J Adolesc Heal. 2021;69(5):754–61.
20. Decker MR, Wood SN, Thiongo M, Byrne ME, Devoto B, Morgan R, et al. Gendered health, economic, social and safety impact of COVID-19 on adolescents and young adults in Nairobi, Kenya. PLoS One. 2021;16(11):e0259583.
21. Zulaika G, Bulbarelli M, Nyothach E, Van Eijk A, Mason L, Fwaya E, et al. Impact of COVID-19 lockdowns on adolescent pregnancy and school dropout among secondary schoolgirls in Kenya. BMJ Glob Heal. 2022;7(1):1–9.
22. DHS. Kenya 2014 Demographic and Health Survey.

For peer review only - http://bmjopen.bmj.com/site/about/guidelines.xhtml

23. National Council for Population and Development. Draft Action Plan for Addressing Adolescent Health and Teenage Pregnancy in Kenya [Internet]. 2021. Available from: https://ncpd.go.ke/wp-content/uploads/2021/10/Draft-Action-Plan-for-Addressing-Adolescent-Health-and-Teenage-Pregnancy-in-Kenya.pdf
24. Quaife M, van Zandvoort K, Gimma A, Shah K, McCreesh N, Prem K, et al. The impact of COVID-19 control measures on social contacts and transmission in Kenyan informal settlements. BMC Med. 2020;18(316):1–11.
25. Yusuf M. Kenya Sees New Surge of Coronavirus After Easing Restrictions. Voices of America. 2020.
26. Magnani R, Sabin K, Saidel T, Heckathorn D. Review of sampling hard-to-reach and hidden populations for HIV surveillance. AIDS. 2005;19(Supplement 2):67–72.
27. International Centre for Reproductive Health-Kenya (ICRHK) & PMA Agile. Nairobi Youth Respondent-Driven Sampling Survey: Final Report. Baltimore, Maryland; 2020.
28. Performance Monitoring for Action (PMA). Performance Monitoring for Action Survey Methodology [Internet]. 2021. Available from: https://www.pmadata.org/data/survey-methodology
29. Karp C, Wood SN, Guiella G, Gichangi P, Bell SO, Anglewicz P, et al. Contraceptive dynamics during COVID-19 in sub- -Saharan Africa: longitudinal evidence from Burkina Faso and Kenya. BMJ Sex Reprod Heal. 2021;0:1–9.
30. Our World in Data. Policy Responses to the Coronavirus Pandemic--Statistics and Research [Internet]. [cited 2022 Jan 26]. Available from: https://ourworldindata.org/policy-responses-covid

For peer review only

For peer review only - http://bmjopen.bmj.com/site/about/guidelines.xhtml

Figure 1. Fertility intentions throughout the pandemic, by gender
Restricted to those with complete survey data at all three time points

For peer review only

For peer review only - http://bmjopen.bmj.com/site/about/guidelines.xhtml

Figure 2. Contraceptive dynamics throughout the pandemic by method effectiveness, by gender
Restricted to those who were sexually active and in need of contraception (not pregnant and did not want a child soon/now or within the next year) at both the time of that survey and the prior survey round, per gender; Method effectiveness: LARC=IUD, implant; short-acting=injectable, pills; coital-dependent=emergency contraception, male condoms, female condoms; other/traditional=rhythm, withdrawal, herbal pill method

For peer review only

Figure 3. Main reason for discontinuation at 12-month (n=143) and 18-month (n=137) follow-up

Pregnancy-related: want to become pregnant; Partner-related: fear of cheating, partner opposed, partner away, infrequent sex, not married; COVID-related: could not go to facility due to COVID-19 restrictions; Access-related: lack of access/too far, cost, preferred method not available, no method available, inconvenient to use, knows no alternate method; Personal concerns about/opposition to contraception: respondent opposed, interferes with body's processes, religious reasons, others opposed, fear of side effects, health concerns

For peer review only

For peer review only - http://bmjopen.bmj.com/site/about/guidelines.xhtml

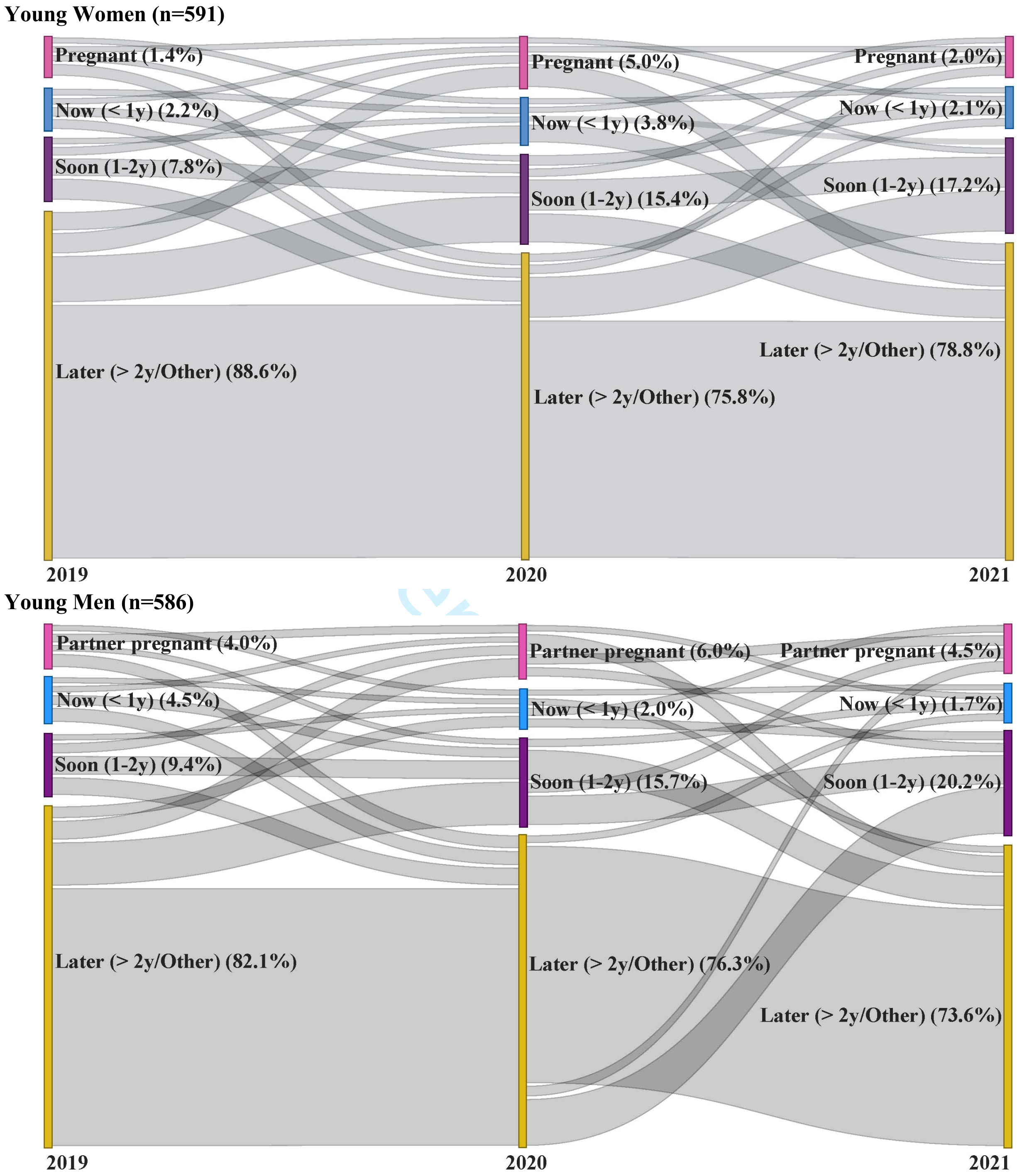
Young Women (n=591)
Pregnant (1.4%)
Now (< 1y) (2.2%)
Soon (1-2y) (7.8%)
Later (> 2y/Other) (88.6%)
Pregnant (5.0%)
Now (< 1y) (3.8%)
Soon (1-2y) (15.4%)
Later (> 2y/Other) (75.8%)
Pregnant (2.0%)
Now (< 1y) (2.1%)
Soon (1-2y) (17.2%)
Later (> 2y/Other) (78.8%)
2019
2020
2021
Young Men (n=586)
Partner pregnant (4.0%)
Now (< 1y) (4.5%)
Soon (1-2y) (9.4%)
Later (> 2y/Other) (82.1%)
Partner pregnant (6.0%)
Now (< 1y) (2.0%)
Soon (1-2y) (15.7%)
Later (> 2y/Other) (76.3%)
Partner pregnant (4.5%)
Now (< 1y) (1.7%)
Soon (1-2y) (20.2%)
Later (> 2y/Other) (73.6%)
2019
2020
2021

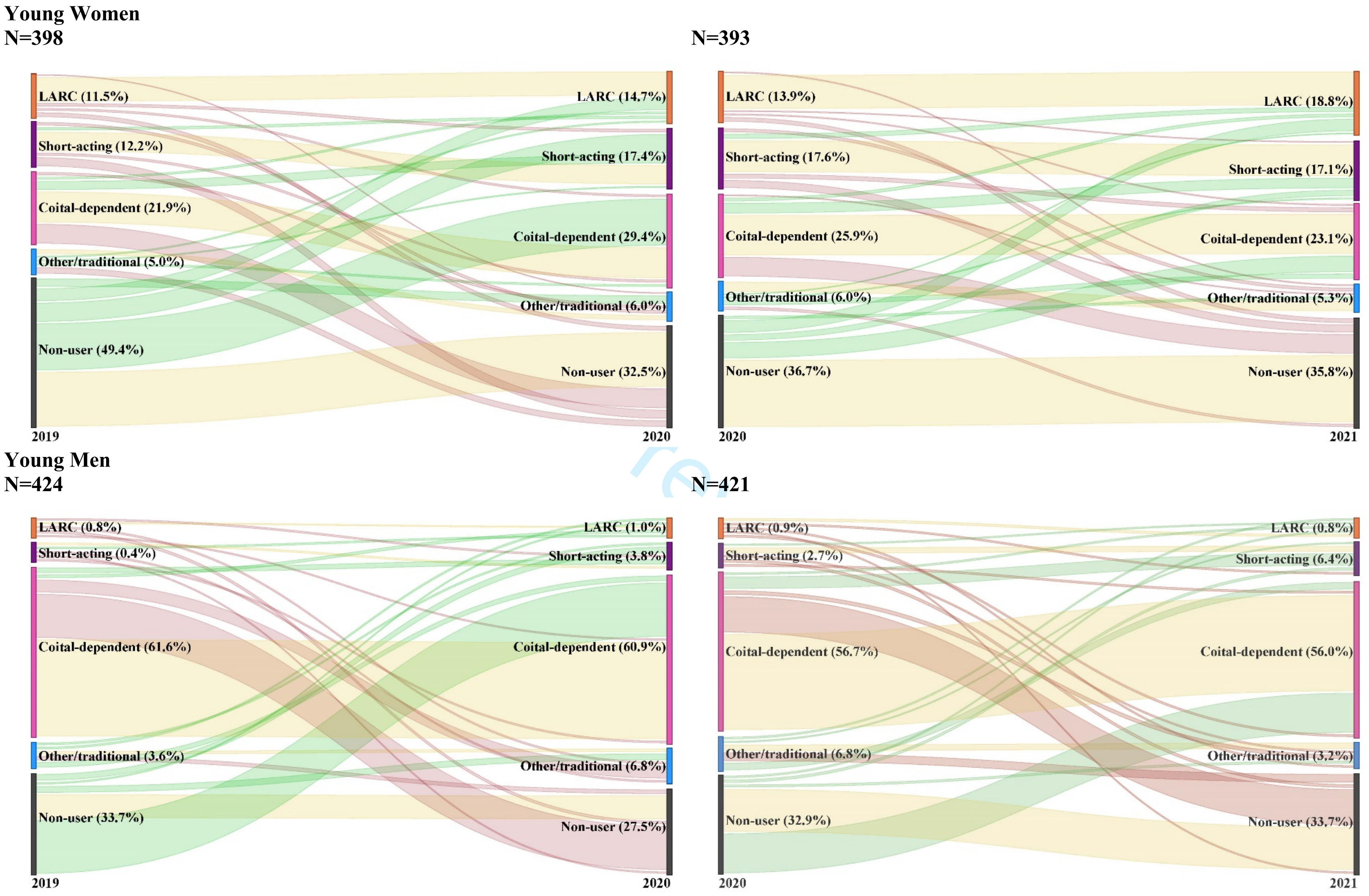
Young Women
N=398
LARC (11.5%)
Short-acting (12.2%)
Coital-dependent (21.9%)
Other/traditional (5.0%)
Non-user (49.4%)
LARC (14.7%)
Short-acting (17.4%)
Coital-dependent (29.4%)
Other/traditional (6.0%)
Non-user (32.5%)
2019
2020
N=393
LARC (13.9%)
Short-acting (17.6%)
Coital-dependent (25.9%)
Other/traditional (6.0%)
Non-user (36.7%)
LARC (18.8%)
Short-acting (17.1%)
Coital-dependent (23.1%)
Other/traditional (5.3%)
Non-user (35.8%)
2020
2021
Young Men
N=424
LARC (0.8%)
Short-acting (0.4%)
Coital-dependent (61.6%)
Other/traditional (3.6%)
Non-user (33.7%)
LARC (1.0%)
Short-acting (3.8%)
Coital-dependent (60.9%)
Other/traditional (6.8%)
Non-user (27.5%)
2019
2020
N=421
LARC (0.9%)
Short-acting (2.7%)
Coital-dependent (56.7%)
Other/traditional (6.8%)
Non-user (32.9%)
LARC (0.8%)
Short-acting (6.4%)
Coital-dependent (56.0%)
Other/traditional (3.2%)
Non-user (33.7%)
2020
2021

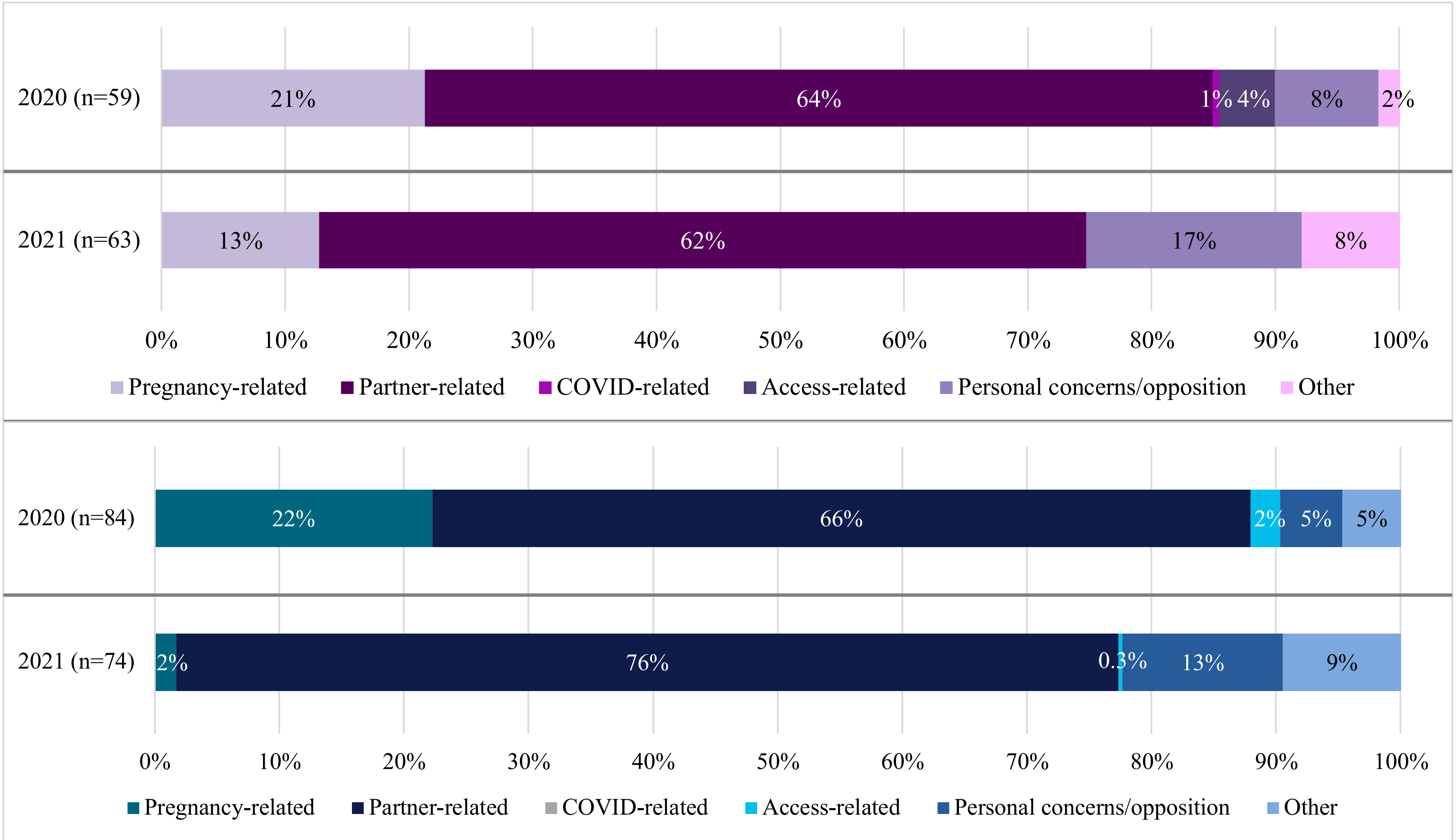
2020 (n=59)
21%
64%
1%
4%
8%
2%
2021 (n=63)
13%
62%
17%
8%
0% 10% 20% 30% 40% 50% 60% 70% 80% 90% 100%
Pregnancy-related
Partner-related
COVID-related
Access-related
Personal concerns/opposition
Other
2020 (n=84)
22%
66%
2%
5%
5%
2021 (n=74)
2%
76%
0.3%
13%
9%
0% 10% 20% 30% 40% 50% 60% 70% 80% 90% 100%
Pregnancy-related
Partner-related
COVID-related
Access-related
Personal concerns/opposition
Other

STROBE Statement—Checklist of items that should be included in reports of ***cohort studies***

	Item No	Recommendation	Page No
**Title and abstract**	1	(*a*) Indicate the study's design with a commonly used term in the title or the abstract	1
		(*b*) Provide in the abstract an informative and balanced summary of what was done and what was found	2
**Introduction**			
Background/rationale	2	Explain the scientific background and rationale for the investigation being reported	4
Objectives	3	State specific objectives, including any prespecified hypotheses	4
**Methods**			
Study design	4	Present key elements of study design early in the paper	5
Setting	5	Describe the setting, locations, and relevant dates, including periods of recruitment, exposure, follow-up, and data collection	5
Participants	6	(*a*) Give the eligibility criteria, and the sources and methods of selection of participants. Describe methods of follow-up	5
		(*b*) For matched studies, give matching criteria and number of exposed and unexposed	NA
Variables	7	Clearly define all outcomes, exposures, predictors, potential confounders, and effect modifiers. Give diagnostic criteria, if applicable	5
Data sources/ measurement	8*	For each variable of interest, give sources of data and details of methods of assessment (measurement). Describe comparability of assessment methods if there is more than one group	5
Bias	9	Describe any efforts to address potential sources of bias	8
Study size	10	Explain how the study size was arrived at	6
Quantitative variables	11	Explain how quantitative variables were handled in the analyses. If applicable, describe which groupings were chosen and why	5
Statistical methods	12	(*a*) Describe all statistical methods, including those used to control for confounding	6
		(*b*) Describe any methods used to examine subgroups and interactions	6
		(*c*) Explain how missing data were addressed	6
		(*d*) If applicable, explain how loss to follow-up was addressed	6
		(*e*) Describe any sensitivity analyses	NA
**Results**			
Participants	13*	(a) Report numbers of individuals at each stage of study—eg numbers potentially eligible, examined for eligibility, confirmed eligible, included in the study, completing follow-up, and analysed	6
		(b) Give reasons for non-participation at each stage	6
		(c) Consider use of a flow diagram	NA
Descriptive data	14*	(a) Give characteristics of study participants (eg demographic, clinical, social) and information on exposures and potential confounders	6
		(b) Indicate number of participants with missing data for each variable of interest	6
		(c) Summarise follow-up time (eg, average and total amount)	5
Outcome data	15*	Report numbers of outcome events or summary measures over time	6

For peer review only

For peer review only - http://bmjopen.bmj.com/site/about/guidelines.xhtml

Main results	16	(*a*) Give unadjusted estimates and, if applicable, confounder-adjusted estimates and their precision (eg, 95% confidence interval). Make clear which confounders were adjusted for and why they were included	7
		(*b*) Report category boundaries when continuous variables were categorized	NA
		(*c*) If relevant, consider translating estimates of relative risk into absolute risk for a meaningful time period	NA
Other analyses	17	Report other analyses done—eg analyses of subgroups and interactions, and sensitivity analyses	7
**Discussion**			
Key results	18	Summarise key results with reference to study objectives	8
Limitations	19	Discuss limitations of the study, taking into account sources of potential bias or imprecision. Discuss both direction and magnitude of any potential bias	8
Interpretation	20	Give a cautious overall interpretation of results considering objectives, limitations, multiplicity of analyses, results from similar studies, and other relevant evidence	8
Generalisability	21	Discuss the generalisability (external validity) of the study results	8
**Other information**			
Funding	22	Give the source of funding and the role of the funders for the present study and, if applicable, for the original study on which the present article is based	10

*Give information separately for exposed and unexposed groups.

Note: An Explanation and Elaboration article discusses each checklist item and gives methodological background and published examples of transparent reporting. The STROBE checklist is best used in conjunction with this article (freely available on the Web sites of PLoS Medicine at http://www.plosmedicine.org/, Annals of Internal Medicine at http://www.annals.org/, and Epidemiology at http://www.epidem.com/). Information on the STROBE Initiative is available at http://www.strobe-statement.org.

For peer review only

BMJ Open

Fertility and Contraceptive Dynamics amidst COVID-19: Who is at Greatest Risk for Unintended Pregnancy among a Cohort of Adolescents and Young Adults in Nairobi, Kenya?

Journal:	*BMJ Open*
Manuscript ID	bmjopen-2022-068689.R1
Article Type:	Original research
Date Submitted by the Author:	27-Feb-2023
Complete List of Authors:	Wood, Shannon; Johns Hopkins University Bloomberg School of Public Health, Department of Population, Family and Reproductive Health<br>Byrne, Meagan E.; Johns Hopkins University Bloomberg School of Public Health, Department of Population, Family and Reproductive Health<br>Thiongo, Mary; International Centre for Reproductive Health Kenya<br>Devoto, Bianca; Johns Hopkins University Bloomberg School of Public Health, Department of Population, Family and Reproductive Health<br>Wamue-Ngare, G; Kenyatta University, Department of Sociology, Gender and Development Studies<br>Decker, Michele; Johns Hopkins University Bloomberg School of Public Health<br>Gichangi, Peter; International Centre for Reproductive Health Kenya
**Primary Subject Heading**:	Global health
Secondary Subject Heading:	Obstetrics and gynaecology, Public health, Reproductive medicine, Sexual health
Keywords:	COVID-19, GYNAECOLOGY, OBSTETRICS, PUBLIC HEALTH, REPRODUCTIVE MEDICINE

SCHOLARONE™
Manuscripts

I, the Submitting Author has the right to grant and does grant on behalf of all authors of the Work (as defined in the below author licence), an exclusive licence and/or a non-exclusive licence for contributions from authors who are: i) UK Crown employees; ii) where BMJ has agreed a CC-BY licence shall apply, and/or iii) in accordance with the terms applicable for US Federal Government officers or employees acting as part of their official duties; on a worldwide, perpetual, irrevocable, royalty-free basis to BMJ Publishing Group Ltd ("BMJ") its licensees and where the relevant Journal is co-owned by BMJ to the co-owners of the Journal, to publish the Work in this journal and any other BMJ products and to exploit all rights, as set out in our licence.

The Submitting Author accepts and understands that any supply made under these terms is made by BMJ to the Submitting Author unless you are acting as an employee on behalf of your employer or a postgraduate student of an affiliated institution which is paying any applicable article publishing charge ("APC") for Open Access articles. Where the Submitting Author wishes to make the Work available on an Open Access basis (and intends to pay the relevant APC), the terms of reuse of such Open Access shall be governed by a Creative Commons licence – details of these licences and which Creative Commons licence will apply to this Work are set out in our licence referred to above.

Other than as permitted in any relevant BMJ Author's Self Archiving Policies, I confirm this Work has not been accepted for publication elsewhere, is not being considered for publication elsewhere and does not duplicate material already published. I confirm all authors consent to publication of this Work and authorise the granting of this licence.

For peer review only

Fertility and Contraceptive Dynamics amidst COVID-19: Who is at Greatest Risk for Unintended Pregnancy among a Cohort of Adolescents and Young Adults in Nairobi, Kenya?

Shannon N. Wood, PhD[1,2]
Meagan E. Byrne, MSPH[1,2]
Mary Thiongo, BS[3]
Bianca Devoto, MSPH[1]
Grace Wamue-Ngare, PhD[4,5]
Michele R. Decker, ScD[1,2]
Peter Gichangi, MD[3,6,7]

[1] Department of Population, Family and Reproductive Health, Johns Hopkins Bloomberg School of Public Health, Baltimore, Maryland, USA.
[2] Bill & Melinda Gates Institute for Population and Reproductive Health, Department of Population, Family and Reproductive Health, Johns Hopkins Bloomberg School of Public Health, Baltimore, Maryland, USA.
[3] International Centre for Reproductive Health-Kenya, Nairobi, Kenya.
[4] Women's Economic Empowerment Hub, Kenyatta University, Nairobi, Kenya.
[5] Department of Sociology, Gender and Development Studies, Kenyatta University, Nairobi, Kenya.
[6] Technical University of Mombasa, Mombasa, Kenya.
[7] Department of Public Health and Primary Care, Faculty of Medicine and Health Sciences, Ghent University, Belgium.

Corresponding Author:
Shannon N. Wood
Johns Hopkins Bloomberg School of Public Health
Department of Population, Family and Reproductive Health
615 N. Wolfe Street Room E4009 Baltimore, MD 21205
swood@jhu.edu
+1 410 502 2303

Word Count: 3,863
Tables: 3
Figures: 3
References: 35
Appendices: 0

For peer review only

Abstract

Objectives: Among youth in Nairobi, we 1) characterized fertility and contraceptive use dynamics by gender; 2) estimated pregnancy prevalence over the pandemic; 3) assessed factors associated with unintended pandemic pregnancy for young women.

Design: Longitudinal analyses utilize cohort data collected at three time points prior to and during the COVID-19 pandemic: June-August 2019 [pre-pandemic], August-October 2020 [12-month follow-up], and April-May 2021 [18-month follow-up].

Setting: Nairobi, Kenya

Participants: At initial cohort recruitment, eligible youth were aged 15-24 years, unmarried, and residing in Nairobi for at least one year. Within timepoint analyses were restricted to participants with survey data per round; trend and prospective analyses were restricted to those with complete data at all three timepoints (n=586 young men, n=589 young women).

Primary and Secondary Outcome Measures: Primary outcomes comprised fertility and contraceptive use for both genders, and pregnancy for young women. Unintended pandemic pregnancy (assessed at 18-month follow-up) was defined as a current or past 6-month pregnancy with intent to delay pregnancy for more than one year at 2020 survey.

Results: While fertility intentions remained stable, contraceptive dynamics varied by gender—young men both adopted and discontinued coital-dependent methods, whereas young women adopted coital-dependent or short-acting methods at 12-month follow-up (2020). Current pregnancy was highest at 2020 (4.8%), and approximately 2% at 2019 and 2021. Unintended pandemic pregnancy prevalence was 6.1%, with increased odds for young women recently married (aOR=3.79; 95% CI=1.83-7.86); recent contraceptive use was protective against unintended pandemic pregnancy (aOR=0.23; 95% CI=0.11-0.47).

Conclusions: Current pregnancy in Nairobi was highest at the height of the COVID-19 pandemic (2020), and subsided to pre-pandemic levels by 2021 data collection, however, requires further monitoring. New marriages posed considerable risk for unintended pandemic pregnancy. Contraceptive use remains a crucial preventive strategy to averting unintended pregnancy, particularly for married young women.

For peer review only

For peer review only - http://bmjopen.bmj.com/site/about/guidelines.xhtml

Strengths and limitations of the study

- The COVID-19 pandemic raised concerns for unintended pregnancy for adolescent girls and young women (AGYW), however, until now, data on this distal outcome had not been available due to time lag.
- To our knowledge this is the first study to longitudinally examine unintended pregnancy incurred during the COVID-19 pandemic among AGYW in sub-Saharan Africa; limitations of retrospective unintended pregnancy assessment are well established, making prospective cohort data uniquely valuable to address this topic.
- The assessment of unintended pandemic pregnancy, while our best estimate, may be subject to measurement error given that data collection was not continually ongoing throughout the pandemic.
- Data were collected immediately prior to the pandemic and at two points after the implementation of COVID-19 restrictions in Nairobi—as Kenya continues to experience COVID-19 infection waves and reinstates mobility restrictions to curb infection spread, ongoing data collection is needed to understand impacts.
- Nairobi is a major urban center and results should not be generalized to all parts of Kenya, where AGYW may face different challenges surrounding sexual and reproductive health.

For peer review only

For peer review only - http://bmjopen.bmj.com/site/about/guidelines.xhtml

Introduction

Adolescent pregnancy is a human rights concern with gendered repercussions that disproportionately impact adolescent girls and young women (AGYW) (1,2). Both intended and unintended pregnancies can alter AGYW's life trajectories, including by decreasing their ability to meet educational, business/financial, and family goals (1). Further, early pregnancy can have negative health effects—adolescents are at risk for increased birth complications, including obstructed labor, fistula, premature delivery, low birth weight, pre-eclampsia/eclampsia, and maternal and perinatal mortality, compared to their older counterparts (1,3). While complete adolescent pregnancy data are difficult to obtain, largely due to underreporting and stigmatization of unintended pregnancies that end in abortions, the evidence is clear that the majority of the global adolescent pregnancy burden is concentrated within sub-Saharan Africa, where adolescent pregnancy rates in most countries exceed 100 per 1,000 women aged 15-19 (4).

Motivations and intentions surrounding adolescent pregnancy are complex and require a socioecological lens (5). Pregnancy intendedness varies substantially by setting and in many contexts, cultural and familial norms may promote early childbearing in lieu of economic and educational attainment (2). Factors linked to adolescent pregnancy in sub-Saharan Africa include low education, lack of familial education, poverty, and limited communication/knowledge surrounding sexual and reproductive health (SRH) (6,7). Ecological data further correlate adolescent pregnancy with high gender inequality at the national level (8). While rural adolescents are generally at heightened risk of pregnancy, largely due to decreased access to SRH services (6,7), urban adolescents have long been a target group for SRH programs given the population's growing size and unique needs (9). Many AGYW migrate to cities to seek out financial opportunities, but instead are met with stigma and shame when seeking healthcare services (10,11); these barriers may be more pronounced for unmarried youth given social norms dictating sex as a means for procreation and stigmatization of sexual activity outside of marriage, and in turn, increase unmet SRH needs (12).

When the COVID-19 outbreak was declared a pandemic by the World Health Organization (WHO) in March 2020, the SRH community was concerned about the negative impact mitigation measures would have on women's SRH, and particularly the SRH of AGYW (13–15). Early COVID-19 projections estimated that 15 million additional unintended pregnancies could occur over one year if COVID-related service disruptions affected 10% of women in need of SRH services in low- and middle-income countries (16). Unintended pregnancy is a distal outcome and requires time and prospective data to accurately assess; thus far, no studies have corroborated these pregnancy projections (17,18). In lieu of unintended pregnancy data, contraceptive use is the most widely used proxy for unintended pregnancy—population-level data from four sub-Saharan African contexts indicate that contraceptive use among women in need increased across settings, contrary to warnings of unintended pregnancy increases (19). While early trends are largely promising, some groups may be at increased risk of unintended pregnancy, namely adolescents and nulliparous women, due to increases in contraceptive need and reductions in contraceptive use during this period (19). Another study utilizing facility data to assess facility admissions and antenatal, delivery, and immunization services reported slight reductions in services (20). Adolescents may further face difficulties procuring contraception during the COVID-19 pandemic given limited household bargaining power and school closures (21,22). Recent causal-comparative research from Western Kenya elucidates increased school dropout and pregnancy among specific to secondary school girls during COVID-19 compared to a pre-COVID-19 cohort (23).

While multiple studies have examined youth contraceptive use and/or sexual activity in light of COVID-19 (17,18,21,22), no study to date has utilized prospective data to examine unintended pregnancy during the COVID-19 pandemic in sub-Saharan Africa for AGYW. Among urban youth in Nairobi, Kenya, this study aimed to 1) characterize fertility and contraceptive use dynamics by gender; 2) estimate pregnancy prevalence for AGYW over the course of the pandemic; and 3) assess factors associated with unintended pandemic pregnancy for young women.

Methods

Study Setting

This study takes place in urban Nairobi, Kenya, where adolescent pregnancy is a country priority (24)—per 2014 Demographic and Health Survey estimates, one-fifth of Kenyan AGYW ages 15-19 were pregnant or already mothers, and approximately half of unmarried, sexually active AGYW reported using a modern method of contraception (25). The COVID-19 pandemic elevated mitigation of adolescent pregnancy within national policies. The first case of COVID-19 in Kenya was detected on March 13, 2020 (26), with school closures, national lockdown, and mandatory curfew immediately following (27). By late 2020 restrictions were eased; however, in summer 2021, caseloads began rising again, prompting additional closures (27). These restrictions, while essential to curbing the spread of COVID-19, were hypothesized to decrease access to essential health services, including SRH services.

For peer review only - http://bmjopen.bmj.com/site/about/guidelines.xhtml

Study Overview

This longitudinal study draws on data collected from the Nairobi Youth Respondent Driven Sampling Survey (YRDSS), a cohort of adolescents and young adults in Nairobi collected at three time points prior to and during the COVID-19 pandemic: June-August 2019 [pre-pandemic], August-October 2020 [12-month follow-up], and April-May 2021 [18-month follow-up].

Briefly, the YRDSS began recruitment in June-August 2019 using respondent-driven sampling (RDS), a chain-based recruitment method that begins with purposefully selected seeds, followed by monitored peer-to-peer coupon distribution (28). At the time of recruitment, eligible youth were aged 15-24 years, unmarried, and residing in Nairobi for at least one year. The 2019 pre-pandemic survey round recruited 1,357 participants; of whom 95% (1,293/1,357) consented for recontact and provided contact information. This cohort was recontacted and surveyed in August-October 2020 (12-month follow-up; n=1,217; 94% retention], and in April-May 2021 (18-month follow-up; n=1,177; 97% retention).

Ethical approval was obtained from the Ethics Review Committee at Kenyatta National Hospital/University of Nairobi (P294/04/2019) and the Institutional Review Board at Johns Hopkins Bloomberg School of Public Health (00009496). Detailed study procedures are outlined elsewhere (29).

Data Collection Procedures

The pre-pandemic [2019] survey was specific to reproductive health needs and family planning behaviors for adolescents and young adults. Data collection occurred in-person with surveys self-administered by participants or administered by trained resident enumerators in case of comprehension or technological difficulty. Data was entered onto tablets and uploaded to a cloud server using Open Data Kit (ODK) software. Surveys were conducted in English or Swahili, based on participant preference.

Given the onset of the COVID-19 pandemic, follow-up surveys at 12-months [2020] and 18-months [2021] were designed specifically to examine the gendered impacts of COVID-19 on youth economic, health, and social experiences, including pandemic impacts on youth contraceptive use and unintended pregnancy. In adherence to COVID-19 restrictions, interviews at these timepoints were conducted by the same resident enumerators via remote phone interviews, with responses recorded using the ODK software.

Measures

Outcomes. Primary outcomes comprised fertility and contraceptive use for both genders, as well as pregnancy for young women.

Prospective fertility intentions were assessed per gender at each of the three time points. Specifically, each participant was asked "How long would you like to wait from now before the birth of a child?" with responses comprising "x months", "x years", "would like to get pregnant soon/now", and "can't get pregnant" (for women) or "can't cause pregnancy" (for men), and "do not want children." For trend analysis, intentions were categorized as "currently pregnant"; "<1 year"; "1-2 years"; ">2 years/other."

Current contraceptive use was assessed per gender at each time point utilizing standard items (30). Contraceptive users were asked current method, and if users reported more than one current method, they were then asked to identify the method they used "mainly" or "most of the time". Responses were split into method effectiveness categories for trend analysis (1-long-acting reversible contraception (LARC): intrauterine device (IUD) and implant; 2-short-acting: injectable and pills; 3-coital-dependent: emergency contraception, male condoms, and female condoms; 4-other/traditional: rhythm, withdrawal, and herbal pill method).

Period and current pregnancy were assessed per time point for young women. At pre-pandemic baseline, young women were asked about ever pregnancy, whereas at 12-month and 18-month follow-up, past 12-month and past 6-month pregnancy were assessed to capture between-survey outcomes. For all young women with a period pregnancy, current pregnancy assessed via single item, "Are you currently pregnant?" A dichotomous variable was then created for the primary outcome of interest, unintended pandemic pregnancy, defined as having a current pregnancy or past 6-month pregnancy at 18-month follow-up survey and wanting to wait more than one year for a pregnancy at last survey round (2020).

For peer review only - http://bmjopen.bmj.com/site/about/guidelines.xhtml

Exposures for unintended pandemic pregnancy: All risk factors for unintended pandemic pregnancy were assessed at 12-month follow-up (2020) survey using standard measures (29,30), and include age (15-19; 20-24); highest level of education completed (no school/primary/post-primary; secondary or higher); subjective household wealth tertile (low; middle; high); employment (someone else vs. self); increased time with partner since COVID-19 restrictions (yes/no); ability to meet basic needs (yes/no); and contraceptive use at 2020 survey. Exposures were dichotomized based on underlying distributions to maximize power for multivariable analyses.

Analytic Sample

Within timepoint analyses were restricted to participants with completed survey data for that round (n=605 young men, n=610 young women at pre-pandemic and 12-month follow-up; n=586 young men, n=591 young women at 18-month follow-up). Trend and prospective analyses were restricted to those with complete data at all three timepoints (n=586 young men, n=589 young women); for contraceptive dynamics, analyses were additionally restricted to those with a need for contraception (i.e., sexually active, not pregnant (if female), and not wanting a child within the next year) at both the time of that survey and the prior survey round. A complete case approach was adopted given <1% missing outcome data (n=2 missing unintended pandemic pregnancy); sample size floats to accommodate small amounts of missing covariate data (<1%).

Statistical Analysis

Descriptive statistics examined characteristics of youth at pre-pandemic, by gender, with design-based F-statistics classifying between-gender differences. Sankey diagrams depicted dynamics in fertility intentions and contraceptive use across the three survey rounds, by gender. Among youth who discontinued their method at 12-month or 18-month follow-up, main reason for discontinuation was examined, per gender and time point. For each round, period and current pregnancy prevalence were calculated for all young women, and among adolescent girls (<18-years-old) and those wishing to avert a pregnancy at last round (i.e., did not want a child within the next year). Lastly, bivariate and multivariable logistic regression examined factors at 12-month follow-up (2020) associated with unintended pandemic pregnancy among young women via glm models with family binomial and link logistic to account for weighing and clustering. Factors with p-values <0.1 from bivariate models were included within multivariable models, withstanding increased time with partner, omitted due to collinearity.

All analyses were conducted using Stata 16 (College Station, TX) with statistical significance set a priori at p<0.05. Sampling weights accommodate the respondent driven sampling study design using RDS-II (Volz-Heckathorn) weights, post-estimation adjustment based on 2014 Kenya Demographic and Health Survey population data (age, sex, education levels), and loss-to-follow-up. All estimates are weighted; statistical testing accounts for clustering among participants recruited by the same recruiter at baseline.

Patient and Public Involvement

This community-engaged study sought public and end-user input at all phases. During the formative research stage prior to the 2019 cohort recruitment, input from community-based, youth-serving organizations informed the study recruitment strategy for feasibility, survey measures and constructs to ensure relevance, and study logistics to maximize participant comfort and confidentiality. All recruitment and procedures were conducted by trained resident enumerators selected from underlying communities, and who provided inputs on measures for clarity and aided in results interpretation. Findings were disseminated in November 2020 and again in September 2021 with stakeholders spanning policy sector, government representatives, elders/faith leaders, community-based organizations, and youth leaders from the study communities.

Results

Characteristics of youth at pre-pandemic, by gender, are outlined in Table 1. The majority of both young men and young women were aged 20-24 (60.7% and 57.2%, respectively) and had completed at least secondary education (62.5% and 57.2%, respectively). While roughly three of four youth had ever had sex, higher proportions of young men reported current involvement in a romantic relationship (young men: 66.5%; young women: 54.5%; p=0.04). Approximately one in ten young men had a partner who had ever been pregnant and 8.0% of young women reported their own pregnancy experience. Future fertility intentions differed by gender, with higher proportions of young men reporting wanting a child soon/now (2.7% young men vs. 0.2% young women) or within the next two years (15.8% young men vs. 11.0% young women; p=0.05). Contraceptive use, method effectiveness, and main current contraceptive method differed significantly by gender. Approximately three-quarters of young men reported current

For peer review only - http://bmjopen.bmj.com/site/about/guidelines.xhtml

contraceptive use (75.2%), compared to half of young women (54.7%; p<0.001). The majority (91.2%) of young men reported male condom use as their main contraceptive method, whereas young women utilized a range of methods (p<0.001).

Table 1. Characteristics of youth at pre-pandemic (2019; n=1,215), weighted

	Young Men (n=605)	Young Women (n=610)	p-value[§]
	n (column %)		
Demographic Characteristics			
Age			0.38
15-19	238 (39.3)	261 (42.8)	
20-24	367 (60.7)	349 (57.2)	
Highest level of education completed			0.62
Post-primary/primary or no school	197 (32.6)	211 (34.7)	
Secondary or higher	408 (67.4)	399 (65.4)	
Relationship and Reproductive Characteristics			
Ever had sex	436 (72.0)	441 (72.3)	0.94
Currently in a romantic/sexual relationship	402 (66.5)	332 (54.5)	**0.04**
Ever pregnant/partner has ever been pregnant	62 (10.2)	49 (8.0)	0.35
Future fertility intentions			**0.05**
Soon/now	16.3 (2.7)	1 (0.2)	
Within the next two years	96 (15.8)	67 (11.0)	
More than two years	410 (67.7)	446 (73.2)	
Does not want children or cannot have children	7 (1.1)	8 (1.4)	
Don't know	76 (12.6)	87 (14.2)	
Contraceptive Characteristics			
Contraceptive use	323 (75.2)	249 (54.7)	**<0.001**
Method effectiveness[±]			**<0.001**
Long-acting reversible contraception	3 (1.0)	48 (19.5)	
Short-acting	2 (0.5)	55 (22.5)	
Barrier or hormonal coital-dependent	286 (93.6)	114 (46.5)	
Other/traditional	15 (4.9)	28 (11.4)	
Main Contraceptive Method[±]			**<0.001**
Implant	3 (1.0)	48 (19.1)	
Coil/IUD	0 (0.0)	7 (2.7)	
Injectable	2 (0.5)	43 (17.0)	
Oral contraceptive pills	0 (0.0)	12 (5.0)	
Emergency contraception	6 (1.9)	24 (9.7)	
Male condom	281 (91.2)	85 (34.0)	
Female condom	0 (0.0)	5 (1.8)	
Cycle beads/Standard Days Method/rhythm	7 (2.4)	12 (4.6)	
Withdrawal	8 (2.5)	10 (3.9)	
Herbal Pill Method	1 (0.5)	5 (2.1)	
Place Obtained Main Contraceptive Method[±]			0.28
Hospital	26 (9.1)	34 (15.1)	
Health Center	89 (30.1)	69 (30.9)	
Clinic/Other Health Provider	26 (9.1)	31 (13.7)	
Pharmacy	91 (31.7)	62 (27.6)	
Other	55 (19.1)	28 (12.7)	

For peer review only - http://bmjopen.bmj.com/site/about/guidelines.xhtml

§p-value from design-based F-statistic; boldface indicates p<0.05
±Among users of contraception
Method effectiveness: LARC=IUD, implant; short-acting=injectable, pills; coital-dependent=emergency contraception, male condoms, female condoms; other/traditional=rhythm, withdrawal, herbal pill method
Bolding indicates p<0.05

Throughout the pandemic, young women's fertility intentions remained relatively stable (Figure 1). Between pre-pandemic and 12-month follow up [2020], largest shifts in fertility intention were seen among women who wanted to wait more than two years at pre-pandemic (88.6%; n=515/591) and then said they wanted a child in 1-2 years at 2020 (11.4%; n=80/591); this trend was similarly observed between 12-month and 18-month follow-up, wherein 9.9% of all women (n=65/591) shifted from the greater than 2 years category to 1-2 years. The second largest shift was seen among young women who wanted to wait more than two years at pre-pandemic, but then reported pregnancy at 12-month [2020] follow-up (4.2%; n=17/591). From 12-month to 18-month follow-up, a corresponding shift from pregnancy to wanting to wait more than two years was reported (4.8%; n=20/591).

Trends in fertility intentions for young men (Figure 1) were similar; however, between 12-month [2020] and 18-month [2021] follow-up, large portions of young men also sought to delay childbearing, with 6.8% changing from wanting a child within 1-2 years to wanting to wait two or more years (n=35/586).

[Figure 1 here]

Contraceptive dynamics throughout the pandemic showed substantial variation by gender (Figure 2). Specifically, young men largely migrated between coital-dependent method use and non-use at both timepoints (including both adoption and discontinuation). Conversely, many young women began as non-users and adopted coital-dependent (33.5%; n=67/192) or short-acting methods (13.3%; n=30/192) at 12-month follow-up [2020]. While some coital-dependent users at 12-month follow-up [2020] discontinued by 18-month follow-up (26.2%; n=28/113) [2021], some non-users also continued to take up coital-dependent (15.2%; n=25/120), short-acting (5.0%; n=6/120), and LARC methods (11.4%; 12/120).

[Figure 2 here]

At both 2020 and 2021 surveys and across genders, the primary reason for contraceptive discontinuation centered around partners, including fear of cheating, partner opposed, partner away, infrequent sex, not married (Young women_{2020}: 64%; Young women_{2021}: 62%; Young men_{2020}: 66%; Young men_{2021} 76%; Figure 3). Additionally, approximately one-fifth of both young women (21%) and young men (22%) at 2020 survey stated discontinuation for pregnancy-related reasons, namely wanting to become pregnant. COVID-related, access-related, and personal concerns were less common.

[Figure 3 here]

Following trends in fertility intentions and contraceptive use, prevalence of pregnancy differed across survey rounds (Table 2). Period prevalence ranged from 31.4% ever pregnant at pre-pandemic (2019) to 6.8% pregnant in the past 6-months at 18-month follow-up (2021). Current pregnancy was highest at the 12-month follow-up survey (2020) at 4.8%, whereas it hovered at approximately two percent at the 2019 and 2021 survey rounds. Unintended pandemic pregnancy (i.e., current pregnancy or past 6-month pregnancy at the 18-month follow-up survey with no intention to get pregnant indicated at the previous survey round) was 6.1%, with prevalence approximately half for girls less than age 18 (3.3%).

For peer review only - http://bmjopen.bmj.com/site/about/guidelines.xhtml

Table 2. Prevalence of pregnancy across survey rounds, weighted

	All AGYW	Adolescent girls (<18 years old at baseline)	Among AGYW wishing to avert a pregnancy at last round§
	% (n)		
Period prevalence			
Pre-pandemic (2019) ever pregnant	31.4 (191)	6.2 (5)	--
12-month follow-up (2020) past 12-month pregnancy	12.5 (76)	1.2 (1)	11.2 (65)
18-month follow-up (2021) past 6-month pregnancy	6.8 (40)	3.3 (1)	6.6 (36)
Current pregnancy			
Pre-pandemic (2019)	1.8 (11)	0.0 (0)	--
12-month follow-up (2020)	4.8 (29)	0.0 (0)	4.6 (27)
18-month follow-up (2021)	2.0 (12)	3.3 (1)	1.6 (9)
Unintended pandemic pregnancy (current pregnancy or past-6-month pregnancy at 18-month follow-up, with no intention to get pregnant at last round (2020)	6.1 (36)	3.3 (1)	--

§Wished to wait for pregnancy >1 year at pre-pandemic (for 12-month follow-up assessment) and >6 months at 12-month follow-up (for 18-month follow-up assessment)

Table 3 presents bivariate and multivariable factors at 12-month follow-up (2020) associated with unintended pandemic pregnancy. Recent marriage (at 2020 survey) was associated with a greater than four-fold increase in unintended pandemic pregnancy in both bivariate (OR=4.89; 95% CI=1.93-12.37; p<0.001) and multivariable models (aOR=4.16; 95% CI=1.65-10.51; p=0.006). Within the bivariate model, increased time with partner since COVID-19 restrictions was also associated with increased risk of unintended pandemic pregnancy (OR=3.69; 95% CI=1.61-8.46; p=0.002), however, was excluded from multivariable models due to collinearity with marital status. Contraceptive use at 2020 survey contributed to an 80% reduction in unintended pandemic pregnancy (aOR=0.21; 95% CI=0.09-0.50; p<0.001). Additionally, within both bivariate models, young women with an inability to meet basic needs displayed a borderline protective effect against unintended pandemic pregnancy (OR=0.47; 95% CI=0.21-1.05; p=0.07); however, this association attenuated when adjusted.

Table 3. Factors at 12-month Follow-up (2020) Associated with Unintended Pandemic Pregnancy for Young Women (n=589)

	Unintended Pandemic Pregnancy Row %	OR (95% CI)	aOR (95% CI)
Factors Assessed at 12-month follow-up (2020)			
Age			
15-19	6.5	Ref	--
20-24	5.9	0.90 (0.37, 2.19)	--
Highest level of education completed			
Post-primary/primary or no school	6.9	Ref	--
Secondary or higher	5.7	0.82 (0.32, 2.08)	--
Household wealth			
Low	6.4	Ref	--
Middle	5.6	0.87 (0.33, 2.34)	--
High	6.2	0.96 (0.34, 2.70)	--
Married			
No	4.5	Ref	ref
Yes	18.9	**4.89 (1.93, 12.37)** ***	**4.16 (1.65, 10.51)** **
Employed			
Student, caregiver, unemployed	5.1	Ref	--
Employed formally or informally	7.2	1.45 (0.60, 3.49)	--
Prime earner in household			

For peer review only - http://bmjopen.bmj.com/site/about/guidelines.xhtml

Someone else	5.9	Ref	--
Self	7.4	1.28 (0.46, 3.57)	--
Increased time with partner since COVID-19 restrictions			
No	3.8	Ref	--
Yes	12.8	**3.69 (1.61, 8.46)** **	--
Ability to meet basic needs			
No	4.1	0.47 (0.21, 1.05) œ	0.54 (0.23, 1.29) œ
Yes	8.4	Ref	ref
Contraceptive use			
No	14.4	Ref	ref
Yes	3.7	**0.23 (0.10, 0.53)** ***	**0.21 (0.09, 0.50)** ***

Increased time with partner omitted from multivariable model due to collinearity
Bolding indicates p<0.05

Discussion

Within a narrative that has largely focused on shifting fertility intentions and contraceptive use amidst the COVID-19 pandemic (16,19,31), these longitudinal results counter those for adult populations (17,18) and indicate that fears of unintended pregnancy for AGYW were indeed warranted. Our results on fertility intentions, contraceptive use, and pregnancy all tell the same story—AGYW in Nairobi, Kenya did not use effective methods of contraception and in turn, experienced unintended pregnancy during the time of highest COVID-19 mitigation measures (2020 survey). Specifically, current pregnancy more than doubled at the 2020 survey wave (4.8%) versus pre-pandemic (1.8%) or 2021 (2.0%) surveys, and the majority of pregnancies at 2020 survey (93%) were unintended. Counter to initial hypotheses, education and economic insecurity did not pose significant threats to unintended pandemic pregnancy—instead, risk among AGYW in Nairobi largely centered on newly initiated marriage.

Notably, recent marriage was the only risk factor associated with increased odds of unintended pandemic pregnancy within the multivariable models, where young women who recently married had a four-fold increase of unintended pandemic pregnancy, as compared to those who remained unmarried (p<0.001). Such findings bolster recent results from national cohorts of adult women predicting increases in unintended pregnancy for nulliparous and young women in Lagos, Nigeria (19); the aforementioned study, however, did not highlight potential increases in unintended pregnancy for young women within Kenya (19). Utilizing longitudinal data from an existing cohort of this hard-to-reach population, our findings elucidate that it may not be age nor parity driving risk for AGYW, but rather power dynamics within a newly formed marriage. Pressures for newly married women may increase risk of unintended pregnancy, both during the COVID-19 pandemic and after, and require further research to understand relationship dynamics for newly married couples. While partner pressures are most prominent, concurrent pressures from family and community members should not be downplayed (32,33), and a socioecological framework should be adopted in future research to fully understand the risks of new marriage for AGYW's SRH. While programs to increase age at marriage and keep girls in school are vital to ensure AGYW's autonomy in the decision to marry and their future livelihoods, interventions to increase relationship quality and help young couples navigate the complexities of this new life stage must be simultaneously prioritized in order for young couples to prosper both individually and jointly.

Shifts in fertility intentions throughout the pandemic largely followed expected patterns and timelines (i.e., women who wanted to wait more than two years at pre-pandemic stated that they then wanted a child within 1-2 years one year later). Some AGYW (4.2%; n=17), however, did report wanting to wait more than two years at pre-pandemic survey but then had an unintended pregnancy at 2020 survey—at 2021 survey, these young women again wanted to wait more than two years for their next pregnancy. While young men's fertility intentions followed similar patterns, between 12-month [2020] and 18-month [2021] follow-up, 6.8% shifted from wanting a child within 1-2 years to wanting to wait two or more years—this shift in intentions could be in part due to the continued economic uncertainty and instability imposed by the COVID-19 pandemic. Results corroborate borderline significant results in multivariable models indicating that AGYW with an inability to meet their basic needs had a 50% reduction in unintended pandemic pregnancy. Ultimately, these results speak to the resilience of young people, as well as their initiative to prepare and financially plan for a pregnancy. Continued investment in youth economic empowerment can ensure young people are able to meet both their SRH and financial goals.

For peer review only - http://bmjopen.bmj.com/site/about/guidelines.xhtml

Moreover, results affirm the effectiveness of primary prevention for unintended pregnancy via contraceptive use. Contraceptive use at previous survey round was the only factor protective against unintended pandemic pregnancy—AGYW who used contraception, regardless of method, had an 80% reduction in unintended pandemic pregnancy, compared to those who were not using contraception at last survey round. Youth must have continued access to non-stigmatizing, youth-friendly, and low-cost SRH services. For AGYW who experience unintended pregnancies, during the COVID-19 pandemic and otherwise, access to postpartum contraception is imperative to deter against rapid repeat pregnancy, while ensuring that timing of future pregnancies aligns with a woman's reproductive intentions.

While early research projected contraceptive use decreases due to COVID-related reasons (16), urban AGYW in Nairobi did not cite inability to go to the facility due to COVID-19 restrictions as their primary reason for discontinuation. At both 2020 and 2021 and across genders, the primary reason for contraceptive discontinuation centered around partners (fear of cheating, partner opposed, partner away, infrequent sex, not married). Notably, the included survey item for COVID-related reasons only measured access challenges via inability to visit facilities due to restrictions. Moreover, respondents were only instructed to select their *primary* reason for discontinuation, though multiple reasons could have been experienced. Recent COVID-19 research has reported negative impacts of COVID-19 on youth relationships (21,22,34), including dissolution of partnerships, increased economic dependency, intimate partner violence, and accelerated marriage timelines; altogether, the current research base describes the difficulty of disentangling reasons for discontinuation, with partnership dynamics potentially serving as a proxy for more distal COVID-related reasons. Partnership and COVID-factors should continue to concurrently be measured in survey research, with further longitudinal research needed to disentangle their full and partial effects on SRH outcomes.

Results should be interpreted considering study limitations. Foremost, data were collected immediately prior to the pandemic and at two points after the implementation of COVID-19 restrictions in Nairobi—as Kenya continues to experience COVID-19 infection waves and reinstates mobility restrictions to curb infection spread, ongoing data collection is needed to understand impacts on AGYW's SRH and well-being. Moreover, the assessment of unintended pandemic pregnancy, while our best estimate, may be subject to measurement error given that data collection was not continually ongoing throughout the pandemic. Namely, some AGYW who were pregnant at the 2020 survey round may have indeed experienced an unintended pandemic pregnancy, although we utilized the 18-month (2021) survey for more accurate estimates. We further recognize that fertility intentions are dynamic; while we utilized prospective intention measures from the most recent previous time point to minimize recall biases associated with intention measurement, we recognize that fertility intentions could shift throughout our short follow-up window. Period pregnancy prevalence estimates utilize different referent timepoints and therefore are not comparable; as such, significance testing between times was not utilized and these data should be interpreted as purely descriptive. Nairobi is a major urban center and results should not be generalized to all parts of Kenya, where AGYW may face different challenges surrounding SRH.

As of February 2023, Kenya has experienced seven COVID-19 infection waves, and the health and economic impacts of these waves may not yet to be fully uncovered; recent notable policy shifts, however, signal positive steps for youth in the country (35). Growing momentum seeks to advance women's safety and reproductive rights, including via affirming access to abortion services, and advocating for the elimination of gender-based violence as a national priority. These prospective data, however, highlight that continued policies are needed to affirm reproductive rights and access to quality contraceptive and SRH care for adolescents and young adults. Foremost, continuous access to quality health education, youth-friendly contraceptive services, and universal health care more broadly is imperative to protect the reproductive autonomy of both young men and young women. Further evidence-informed policy efforts should center on dismantling fertility pressures within the community and partner dyad, particularly for AGYW transitioning from dating to marital relationships and still seeking to avert pregnancy. Additional data collection in this arena can aid in monitoring fertility pressures, contraceptive access and use, and quality of care to better understand the continued SRH needs of this vulnerable group. Such youth-friendly policies are crucial for advancing adolescent and young adult SRH both in times of global public health crises and in times of relative stability.

For peer review only - http://bmjopen.bmj.com/site/about/guidelines.xhtml

Funding: This work was supported, in whole, by the Bill & Melinda Gates Foundation [010481]. Under the grant conditions of the Foundation, a Creative Commons Attribution 4.0 Generic License has already been assigned to the Author Accepted Manuscript version that might arise from this submission. The funders had no role in the study design, collection, analysis, and interpretation of data, in writing of the report, or in the decision to submit for publication.

Contributors: Shannon N. Wood contributed to study design, literature search, data analysis, data interpretation, and writing. Meagan E. Byrne contributed to study design, literature search, data analysis, figures, data interpretation, and writing. Mary Thiongo contributed to study design, data interpretation, and writing. Bianca Devoto contributed to study design, data interpretation, and writing. Grace Wamue-Ngare contributed to data interpretation and writing. Michele R. Decker contributed to study design, data interpretation, and writing. Peter Gichangi contributed to study design, data collection, data interpretation, and writing. All authors read and approved the final version of this manuscript.

Ethics Approval: Ethical approval was obtained from the Ethics Review Committee at Kenyatta National Hospital/University of Nairobi (P294/04/2019) and the Institutional Review Board at Johns Hopkins Bloomberg School of Public Health (00009496).

Declaration of interests: No conflicts of interest to disclose.

Acknowledgements: We acknowledge Dr. Caroline Moreau for her assistance in defining our analytic sample and outcome variables.

Data Sharing: Data are available upon request from www.pmadata.org.

For peer review only

References

1. UNFPA. Motherhood in Childhood [Internet]. The State of the World Population 2013. 2013. Available from: http://www.unfpa.org/sites/default/files/pub-pdf/EN-SWOP2013.pdf
2. Chandra-Mouli V, Camacho AV, Michaud PA. WHO guidelines on preventing early pregnancy and poor reproductive outcomes among adolescents in developing countries. J Adolesc Heal. 2013;52(5):517–22.
3. Grønvik T, Sandøy IF. Complications associated with adolescent childbearing in Sub-Saharan Africa: A systematic literature review and meta-analysis. PLoS One. 2018;13(9):1–21.
4. Sedgh G, Finer LB, Bankole A, Eilers MA, Singh S. Adolescent Pregnancy, Birth, and Abortion Rates Across Countries: Levels and Recent Trends. J Adolesc Heal [Internet]. 2015;56(2):223–30. Available from: http://dx.doi.org/10.1016/j.jadohealth.2014.09.007
5. Blum RW, Bastos FI, Kabiru CW, Le LC. Adolescent health in the 21st century. Lancet. 2012;379(9826):1567–8.
6. Kassa GM, Arowojolu AO, Odukogbe AA, Yalew AW. Prevalence and determinants of adolescent pregnancy in Africa: A systematic review and Meta-analysis 11 Medical and Health Sciences 1117 Public Health and Health Services. Reprod Health. 2018;15(1):1–17.
7. Yakubu I, Salisu WJ. Determinants of adolescent pregnancy in sub-Saharan Africa: A systematic review. Reprod Health. 2018;15(1).
8. Decker MR, Kalamar A, Tunçalp Ö, Hindin MJ. Early adolescent childbearing in low- and middle-income countries: Associations with income inequity, human development and gender equality. Health Policy Plan. 2017;32(2):277–82.
9. Melesse DY, Mutua MK, Choudhury A, Wado YD, Faye CM, Neal S, et al. Adolescent sexual and reproductive health in sub-Saharan Africa: Who is left behind? BMJ Glob Heal. 2020;5(e002231).
10. Stidham Hall K, Manu A, Morhe E, Dalton V, Challa S, Loll D, et al. Bad girl and unmet family planning need among Sub-Saharan African adolescents: the role of sexual and reproductive health stigma. Qual Res Med Healthc. 2018;2(1):55–64.
11. Wood K, Jewkes R. Blood Blockages and Scolding Nurses: Barriers to Adolescent Contraceptive Use in South Africa. Reprod Health Matters. 2006;14(27):109–18.
12. Chandra-Mouli V, McCarraher DR, Phillips SJ, Williamson NE, Hainsworth G. Contraception for adolescents in low and middle income countries: Needs, barriers, and access. Reprod Health. 2014;11(1):1–8.
13. Hussein J. COVID-19: What implications for sexual and reproductive health and rights globally? Sex Reprod Heal Matters. 2020;28(1).
14. Thomas AB, Yaya S. Looking ahead in the COVID‑19 pandemic : emerging lessons learned for sexual and reproductive health services in low‑ and middle‑ income countries. Reprod Health. 2021;4:1–8.
15. Marie Stopes International. COVID-19 threatens women's health [Internet]. 2020. Available from: https://mariestopes-us.org/2020/covid-19-threatens-womens-health/
16. Riley T, Sully E, Ahmed Z, Biddlecom A. Estimates of the Potential Impact of the COVID-19 Pandemic on Sexual and Reproductive Health In Low- and Middle-Income Countries. Int Perspect Sex Reprod Health. 2020;46:73–6.
17. Moreau C, Karp C, Wood S, Williams K, Olaolorun FM, Akilimali P, et al. Trends in fertility intentions and contraceptive practices in the context of COVID-19 in sub-Saharan Africa: insights from four national and regional population-based cohorts. BMJ Open. 2023;13(1):e062385.
18. Backhaus A. Pregnancies and contraceptive use in four African countries during the COVID-19 pandemic. Vienna Yearb Popul Res. 2022;20:1–18.
19. Wood SN, Karp C, OlaOlorun F, Akilimali P, Guiella G, Gichangi P, et al. Trends in the need for and use of contraception before and during COVID-19 in four sub-Saharan African geographies: results from population-based national or regional cohort surveys. Lancet Glob Heal. 2021;9:e793-801.
20. Amouzou A, Maïga A, Faye CM, Chakwera S, Melesse DY, Mutua MK, et al. Health service utilisation during the COVID-19 pandemic in sub-Saharan Africa in 2020: A multicountry empirical assessment with a focus on maternal, newborn and child health services. BMJ Glob Heal. 2022;7(5).
21. Karp C, Moreau C, Sheehy G, Anjur-Dietrich S, Mbushi F, Muluve E, et al. Youth Relationships in the Era of COVID-19: A Mixed-Methods Study Among Adolescent Girls and Young Women in Kenya. J Adolesc Heal. 2021;69(5):754–61.
22. Decker MR, Wood SN, Thiongo M, Byrne ME, Devoto B, Morgan R, et al. Gendered health, economic, social and safety impact of COVID-19 on adolescents and young adults in Nairobi, Kenya. PLoS One.

For peer review only - http://bmjopen.bmj.com/site/about/guidelines.xhtml

2021;16(11):e0259583.
23. Zulaika G, Bulbarelli M, Nyothach E, Van Eijk A, Mason L, Fwaya E, et al. Impact of COVID-19 lockdowns on adolescent pregnancy and school dropout among secondary schoolgirls in Kenya. BMJ Glob Heal. 2022;7(1):1–9.
24. National Council for Population and Development. Draft Action Plan for Addressing Adolescent Health and Teenage Pregnancy in Kenya [Internet]. 2021. Available from: https://ncpd.go.ke/wp-content/uploads/2021/10/Draft-Action-Plan-for-Addressing-Adolescent-Health-and-Teenage-Pregnancy-in-Kenya.pdf
25. DHS. Kenya 2014 Demographic and Health Survey.
26. Quaife M, van Zandvoort K, Gimma A, Shah K, McCreesh N, Prem K, et al. The impact of COVID-19 control measures on social contacts and transmission in Kenyan informal settlements. BMC Med. 2020;18(316):1–11.
27. Yusuf M. Kenya Sees New Surge of Coronavirus After Easing Restrictions [Internet]. Voices of America. 2020 [cited 2020 Oct 19]. Available from: https://www.voanews.com/a/covid-19-pandemic_kenya-sees-new-surge-coronavirus-after-easing-restrictions/6197319.html
28. Magnani R, Sabin K, Saidel T, Heckathorn D. Review of sampling hard-to-reach and hidden populations for HIV surveillance. AIDS. 2005;19(Supplement 2):67–72.
29. International Centre for Reproductive Health-Kenya (ICRHK) & PMA Agile. Nairobi Youth Respondent-Driven Sampling Survey: Final Report. Baltimore, Maryland; 2020.
30. Performance Monitoring for Action (PMA). Performance Monitoring for Action Survey Methodology [Internet]. 2021. Available from: https://www.pmadata.org/data/survey-methodology
31. Karp C, Wood SN, Guiella G, Gichangi P, Bell SO, Anglewicz P, et al. Contraceptive dynamics during COVID-19 in sub- -Saharan Africa: longitudinal evidence from Burkina Faso and Kenya. BMJ Sex Reprod Heal. 2021;0:1–9.
32. Karp C, Wood SN, Hadiza G, Kibira SPS, Makumbi F, Omoluabi E, et al. "I am the master key that opens and locks": Presentation and application of a conceptual framework for women's and girls' empowerment in reproductive health. Soc Sci Med. 2020;258(113086).
33. Wood SN, Kennedy SR, Akumu I, Tallam C, Asira B, Hameeduddin Z, et al. Correlates and contributors of reproductive coercion across the socioecological framework among intimate partner violence survivors in Nairobi, Kenya. J Fam Violence. 2022;
34. Decker MR, Bevilacqua K, Wood SN, Ngare GW, Thiongo M, Byrne ME, et al. Gender-based violence during COVID-19 among adolescent girls and young women in Nairobi, Kenya: a mixed-methods prospective study over 18 months. BMJ Glob Heal. 2022;7(2):1–14.
35. Our World in Data. Policy Responses to the Coronavirus Pandemic--Statistics and Research [Internet]. [cited 2022 Jan 26]. Available from: https://ourworldindata.org/policy-responses-covid

For peer review only

1 **Figure 1. Fertility intentions throughout the pandemic, by gender**
2 Restricted to those with complete survey data at all three time points

For peer review only

For peer review only - http://bmjopen.bmj.com/site/about/guidelines.xhtml

Figure 2. Contraceptive dynamics throughout the pandemic by method effectiveness, by gender

Restricted to those who were sexually active and in need of contraception (not pregnant and did not want a child soon/now or within the next year) at both the time of that survey and the prior survey round, per gender; Method effectiveness: LARC=IUD, implant; short-acting=injectable, pills; coital-dependent=emergency contraception, male condoms, female condoms; other/traditional=rhythm, withdrawal, herbal pill method

For peer review only

Figure 3. Main reason for discontinuation at 12-month (n=143) and 18-month (n=137) follow-up
Pregnancy-related: want to become pregnant; Partner-related: fear of cheating, partner opposed, partner away, infrequent sex, not married; COVID-related: could not go to facility due to COVID-19 restrictions; Access-related: lack of access/too far, cost, preferred method not available, no method available, inconvenient to use, knows no alternate method; Personal concerns about/opposition to contraception: respondent opposed, interferes with body's processes, religious reasons, others opposed, fear of side effects, health concerns

For peer review only

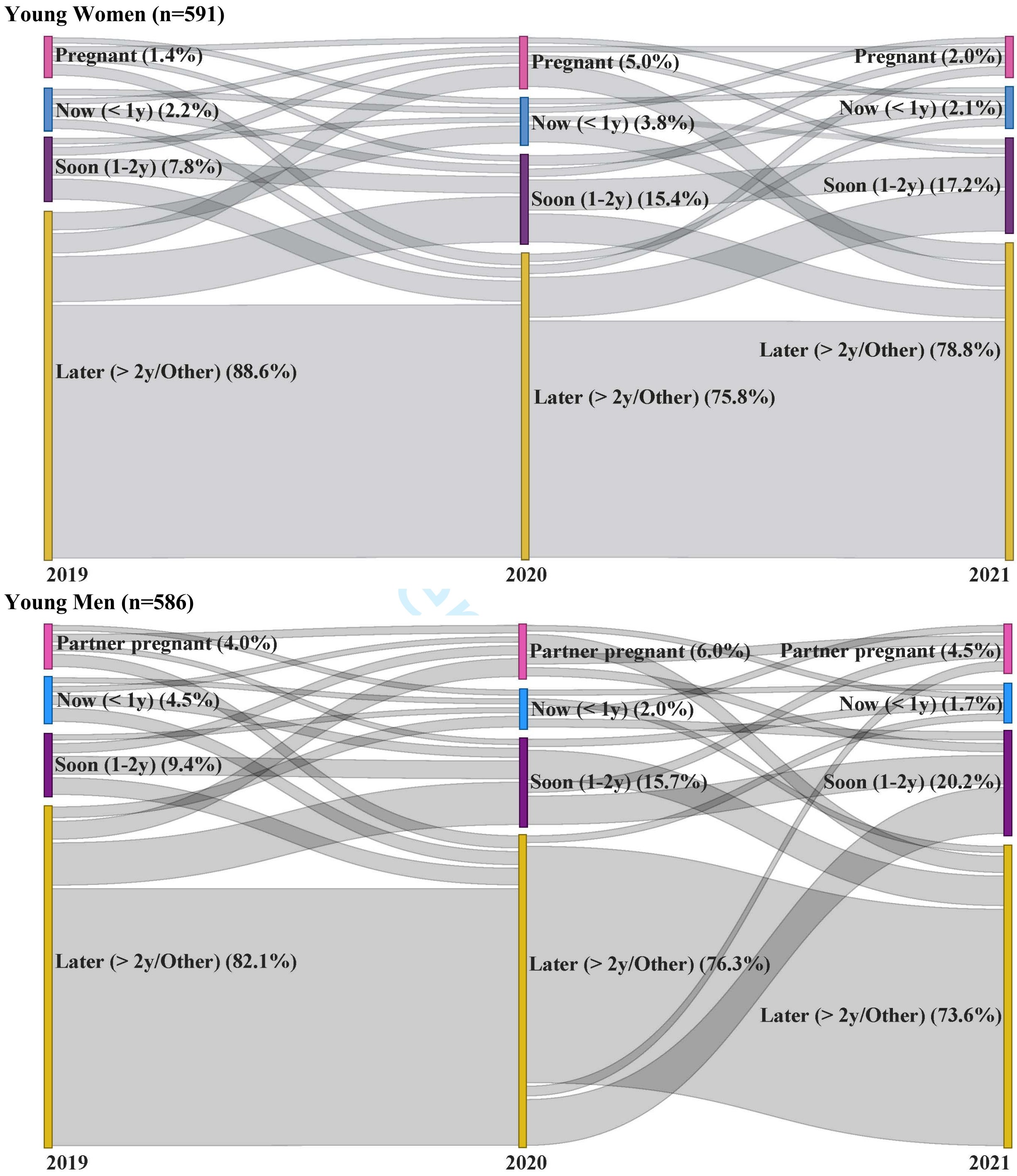
Young Women (n=591)
Pregnant (1.4%)
Now (< 1y) (2.2%)
Soon (1-2y) (7.8%)
Later (> 2y/Other) (88.6%)
Pregnant (5.0%)
Now (< 1y) (3.8%)
Soon (1-2y) (15.4%)
Later (> 2y/Other) (75.8%)
Pregnant (2.0%)
Now (< 1y) (2.1%)
Soon (1-2y) (17.2%)
Later (> 2y/Other) (78.8%)
2019
2020
2021
Young Men (n=586)
Partner pregnant (4.0%)
Now (< 1y) (4.5%)
Soon (1-2y) (9.4%)
Later (> 2y/Other) (82.1%)
Partner pregnant (6.0%)
Now (< 1y) (2.0%)
Soon (1-2y) (15.7%)
Later (> 2y/Other) (76.3%)
Partner pregnant (4.5%)
Now (< 1y) (1.7%)
Soon (1-2y) (20.2%)
Later (> 2y/Other) (73.6%)
2019
2020
2021

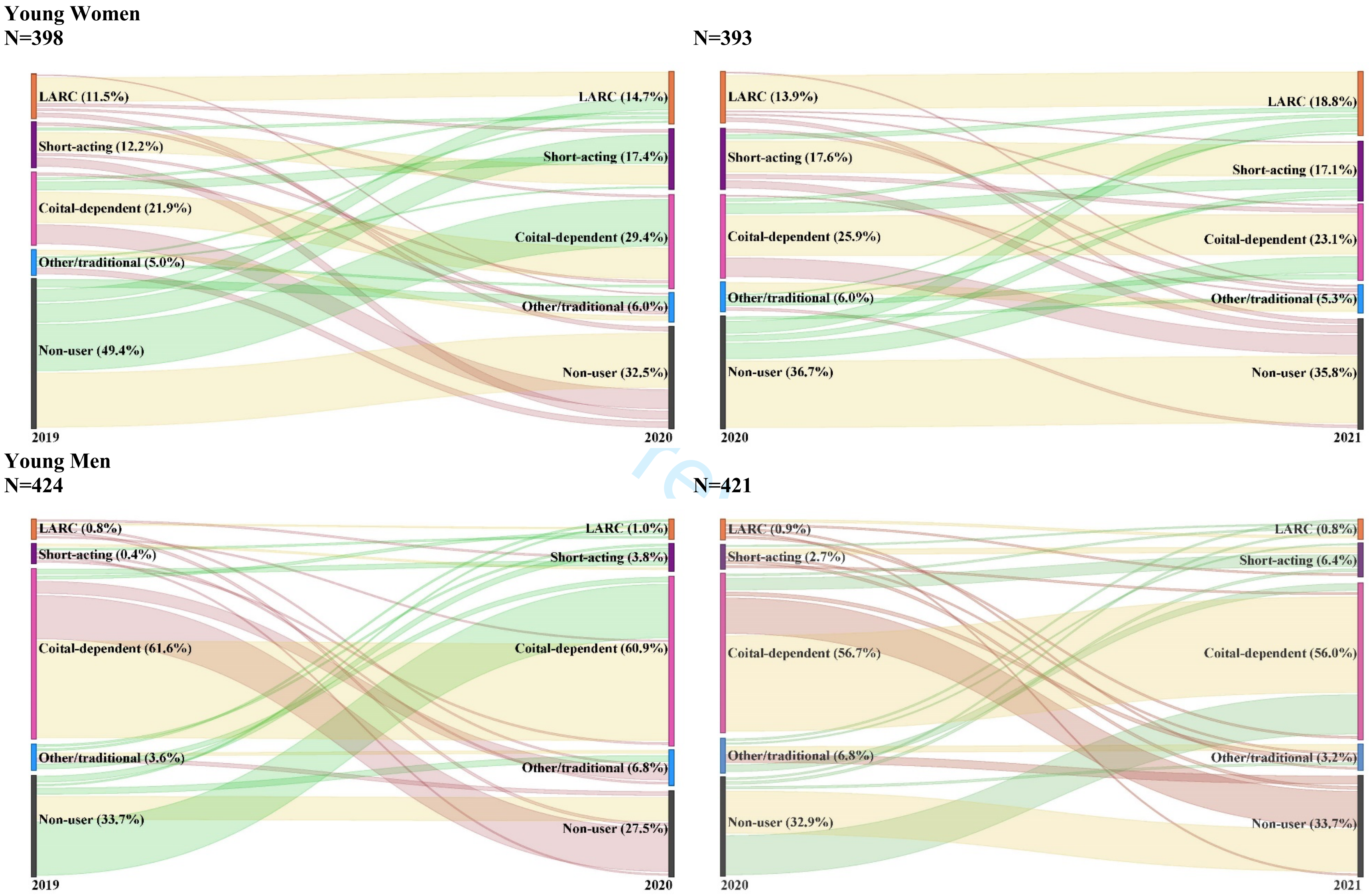
Young Women
N=398
LARC (11.5%)
Short-acting (12.2%)
Coital-dependent (21.9%)
Other/traditional (5.0%)
Non-user (49.4%)
LARC (14.7%)
Short-acting (17.4%)
Coital-dependent (29.4%)
Other/traditional (6.0%)
Non-user (32.5%)
2019
2020
N=393
LARC (13.9%)
Short-acting (17.6%)
Coital-dependent (25.9%)
Other/traditional (6.0%)
Non-user (36.7%)
LARC (18.8%)
Short-acting (17.1%)
Coital-dependent (23.1%)
Other/traditional (5.3%)
Non-user (35.8%)
2020
2021
Young Men
N=424
LARC (0.8%)
Short-acting (0.4%)
Coital-dependent (61.6%)
Other/traditional (3.6%)
Non-user (33.7%)
LARC (1.0%)
Short-acting (3.8%)
Coital-dependent (60.9%)
Other/traditional (6.8%)
Non-user (27.5%)
2019
2020
N=421
LARC (0.9%)
Short-acting (2.7%)
Coital-dependent (56.7%)
Other/traditional (6.8%)
Non-user (32.9%)
LARC (0.8%)
Short-acting (6.4%)
Coital-dependent (56.0%)
Other/traditional (3.2%)
Non-user (33.7%)
2020
2021

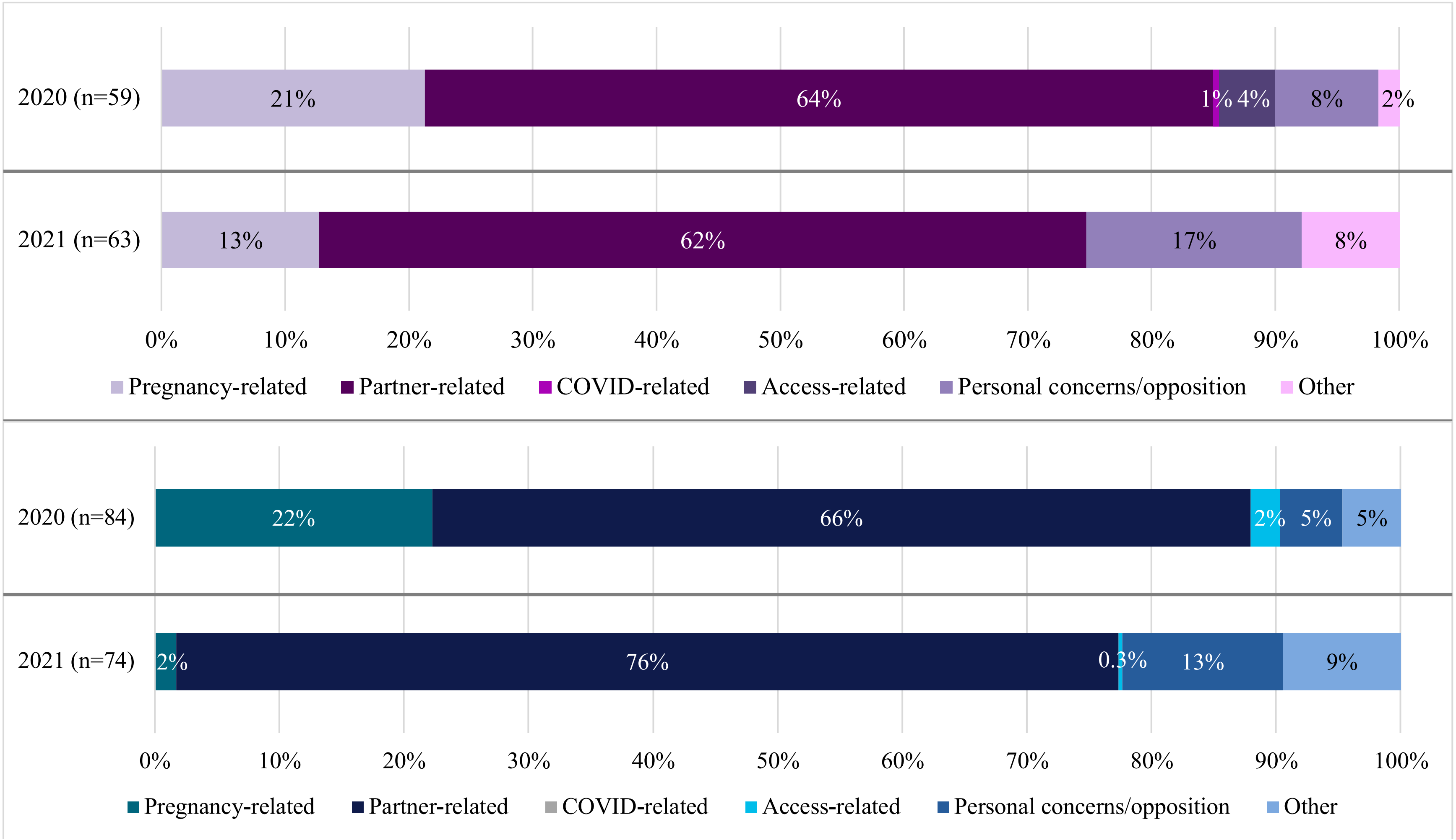
2020 (n=59)
21%
64%
1%
4%
8%
2%
2021 (n=63)
13%
62%
17%
8%
0% 10% 20% 30% 40% 50% 60% 70% 80% 90% 100%
Pregnancy-related
Partner-related
COVID-related
Access-related
Personal concerns/opposition
Other
2020 (n=84)
22%
66%
2%
5%
5%
2021 (n=74)
2%
76%
0.3%
13%
9%
0% 10% 20% 30% 40% 50% 60% 70% 80% 90% 100%
Pregnancy-related
Partner-related
COVID-related
Access-related
Personal concerns/opposition
Other

STROBE Statement—Checklist of items that should be included in reports of ***cohort studies***

	Item No	Recommendation	Page No
**Title and abstract**	1	(*a*) Indicate the study's design with a commonly used term in the title or the abstract	1
		(*b*) Provide in the abstract an informative and balanced summary of what was done and what was found	2
**Introduction**			
Background/rationale	2	Explain the scientific background and rationale for the investigation being reported	4
Objectives	3	State specific objectives, including any prespecified hypotheses	4
**Methods**			
Study design	4	Present key elements of study design early in the paper	5
Setting	5	Describe the setting, locations, and relevant dates, including periods of recruitment, exposure, follow-up, and data collection	5
Participants	6	(*a*) Give the eligibility criteria, and the sources and methods of selection of participants. Describe methods of follow-up	5
		(*b*) For matched studies, give matching criteria and number of exposed and unexposed	NA
Variables	7	Clearly define all outcomes, exposures, predictors, potential confounders, and effect modifiers. Give diagnostic criteria, if applicable	5
Data sources/ measurement	8*	For each variable of interest, give sources of data and details of methods of assessment (measurement). Describe comparability of assessment methods if there is more than one group	5
Bias	9	Describe any efforts to address potential sources of bias	8
Study size	10	Explain how the study size was arrived at	6
Quantitative variables	11	Explain how quantitative variables were handled in the analyses. If applicable, describe which groupings were chosen and why	5
Statistical methods	12	(*a*) Describe all statistical methods, including those used to control for confounding	6
		(*b*) Describe any methods used to examine subgroups and interactions	6
		(*c*) Explain how missing data were addressed	6
		(*d*) If applicable, explain how loss to follow-up was addressed	6
		(*e*) Describe any sensitivity analyses	NA
**Results**			
Participants	13*	(a) Report numbers of individuals at each stage of study—eg numbers potentially eligible, examined for eligibility, confirmed eligible, included in the study, completing follow-up, and analysed	6
		(b) Give reasons for non-participation at each stage	6
		(c) Consider use of a flow diagram	NA
Descriptive data	14*	(a) Give characteristics of study participants (eg demographic, clinical, social) and information on exposures and potential confounders	6
		(b) Indicate number of participants with missing data for each variable of interest	6
		(c) Summarise follow-up time (eg, average and total amount)	5
Outcome data	15*	Report numbers of outcome events or summary measures over time	6

For peer review only

Main results	16	(*a*) Give unadjusted estimates and, if applicable, confounder-adjusted estimates and their precision (eg, 95% confidence interval). Make clear which confounders were adjusted for and why they were included	7
		(*b*) Report category boundaries when continuous variables were categorized	NA
		(*c*) If relevant, consider translating estimates of relative risk into absolute risk for a meaningful time period	NA
Other analyses	17	Report other analyses done—eg analyses of subgroups and interactions, and sensitivity analyses	7
**Discussion**			
Key results	18	Summarise key results with reference to study objectives	8
Limitations	19	Discuss limitations of the study, taking into account sources of potential bias or imprecision. Discuss both direction and magnitude of any potential bias	8
Interpretation	20	Give a cautious overall interpretation of results considering objectives, limitations, multiplicity of analyses, results from similar studies, and other relevant evidence	8
Generalisability	21	Discuss the generalisability (external validity) of the study results	8
**Other information**			
Funding	22	Give the source of funding and the role of the funders for the present study and, if applicable, for the original study on which the present article is based	10

*Give information separately for exposed and unexposed groups.

Note: An Explanation and Elaboration article discusses each checklist item and gives methodological background and published examples of transparent reporting. The STROBE checklist is best used in conjunction with this article (freely available on the Web sites of PLoS Medicine at http://www.plosmedicine.org/, Annals of Internal Medicine at http://www.annals.org/, and Epidemiology at http://www.epidem.com/). Information on the STROBE Initiative is available at http://www.strobe-statement.org.

For peer review only

For peer review only - http://bmjopen.bmj.com/site/about/guidelines.xhtml

BMJ Open

Fertility and Contraceptive Dynamics amidst COVID-19: Who is at Greatest Risk for Unintended Pregnancy among a Cohort of Adolescents and Young Adults in Nairobi, Kenya?

Journal:	*BMJ Open*
Manuscript ID	bmjopen-2022-068689.R2
Article Type:	Original research
Date Submitted by the Author:	17-Apr-2023

Complete List of Authors:	Wood, Shannon; Johns Hopkins University Bloomberg School of Public Health, Department of Population, Family and Reproductive Health<br>Byrne, Meagan E.; Johns Hopkins University Bloomberg School of Public Health, Department of Population, Family and Reproductive Health<br>Thiongo, Mary; International Centre for Reproductive Health Kenya<br>Devoto, Bianca; Johns Hopkins University Bloomberg School of Public Health, Department of Population, Family and Reproductive Health<br>Wamue-Ngare, G; Kenyatta University, Department of Sociology, Gender and Development Studies<br>Decker, Michele; Johns Hopkins University Bloomberg School of Public Health<br>Gichangi, Peter; International Centre for Reproductive Health Kenya

**Primary Subject Heading**:	Global health
Secondary Subject Heading:	Obstetrics and gynaecology, Public health, Reproductive medicine, Sexual health
Keywords:	COVID-19, GYNAECOLOGY, OBSTETRICS, PUBLIC HEALTH, REPRODUCTIVE MEDICINE

SCHOLARONE™
Manuscripts

I, the Submitting Author has the right to grant and does grant on behalf of all authors of the Work (as defined in the below author licence), an exclusive licence and/or a non-exclusive licence for contributions from authors who are: i) UK Crown employees; ii) where BMJ has agreed a CC-BY licence shall apply, and/or iii) in accordance with the terms applicable for US Federal Government officers or employees acting as part of their official duties; on a worldwide, perpetual, irrevocable, royalty-free basis to BMJ Publishing Group Ltd ("BMJ") its licensees and where the relevant Journal is co-owned by BMJ to the co-owners of the Journal, to publish the Work in this journal and any other BMJ products and to exploit all rights, as set out in our licence.

The Submitting Author accepts and understands that any supply made under these terms is made by BMJ to the Submitting Author unless you are acting as an employee on behalf of your employer or a postgraduate student of an affiliated institution which is paying any applicable article publishing charge ("APC") for Open Access articles. Where the Submitting Author wishes to make the Work available on an Open Access basis (and intends to pay the relevant APC), the terms of reuse of such Open Access shall be governed by a Creative Commons licence – details of these licences and which Creative Commons licence will apply to this Work are set out in our licence referred to above.

Other than as permitted in any relevant BMJ Author's Self Archiving Policies, I confirm this Work has not been accepted for publication elsewhere, is not being considered for publication elsewhere and does not duplicate material already published. I confirm all authors consent to publication of this Work and authorise the granting of this licence.

For peer review only

Fertility and Contraceptive Dynamics amidst COVID-19: Who is at Greatest Risk for Unintended Pregnancy among a Cohort of Adolescents and Young Adults in Nairobi, Kenya?

Shannon N. Wood, PhD[1,2]
Meagan E. Byrne, MSPH[1,2]
Mary Thiongo, BS[3]
Bianca Devoto, MSPH[1]
Grace Wamue-Ngare, PhD[4,5]
Michele R. Decker, ScD[1,2]
Peter Gichangi, MD[3,6,7]

[1] Department of Population, Family and Reproductive Health, Johns Hopkins Bloomberg School of Public Health, Baltimore, Maryland, USA.
[2] Bill & Melinda Gates Institute for Population and Reproductive Health, Department of Population, Family and Reproductive Health, Johns Hopkins Bloomberg School of Public Health, Baltimore, Maryland, USA.
[3] International Centre for Reproductive Health-Kenya, Nairobi, Kenya.
[4] Women's Economic Empowerment Hub, Kenyatta University, Nairobi, Kenya.
[5] Department of Sociology, Gender and Development Studies, Kenyatta University, Nairobi, Kenya.
[6] Technical University of Mombasa, Mombasa, Kenya.
[7] Department of Public Health and Primary Care, Faculty of Medicine and Health Sciences, Ghent University, Belgium.

Corresponding Author:
Shannon N. Wood
Johns Hopkins Bloomberg School of Public Health
Department of Population, Family and Reproductive Health
615 N. Wolfe Street Room E4009 Baltimore, MD 21205
swood@jhu.edu
+1 410 502 2303

Word Count: 3,863
Tables: 3
Figures: 3
References: 35
Appendices: 0

For peer review only

Abstract

Objectives: Among youth in Nairobi, we 1) characterized fertility and contraceptive use dynamics by gender; 2) estimated pregnancy prevalence over the pandemic; 3) assessed factors associated with unintended pandemic pregnancy for young women.

Design: Longitudinal analyses utilize cohort data collected at three time points prior to and during the COVID-19 pandemic: June-August 2019 [pre-pandemic], August-October 2020 [12-month follow-up], and April-May 2021 [18-month follow-up].

Setting: Nairobi, Kenya

Participants: At initial cohort recruitment, eligible youth were aged 15-24 years, unmarried, and residing in Nairobi for at least one year. Within timepoint analyses were restricted to participants with survey data per round; trend and prospective analyses were restricted to those with complete data at all three timepoints (n=586 young men, n=589 young women).

Primary and Secondary Outcome Measures: Primary outcomes comprised fertility and contraceptive use for both genders, and pregnancy for young women. Unintended pandemic pregnancy (assessed at 18-month follow-up) was defined as a current or past 6-month pregnancy with intent to delay pregnancy for more than one year at 2020 survey.

Results: While fertility intentions remained stable, contraceptive dynamics varied by gender—young men both adopted and discontinued coital-dependent methods, whereas young women adopted coital-dependent or short-acting methods at 12-month follow-up (2020). Current pregnancy was highest at 2020 (4.8%), and approximately 2% at 2019 and 2021. Unintended pandemic pregnancy prevalence was 6.1%, with increased odds for young women recently married (aOR=3.79; 95% CI=1.83-7.86); recent contraceptive use was protective against unintended pandemic pregnancy (aOR=0.23; 95% CI=0.11-0.47).

Conclusions: Current pregnancy in Nairobi was highest at the height of the COVID-19 pandemic (2020), and subsided to pre-pandemic levels by 2021 data collection, however, requires further monitoring. New marriages posed considerable risk for unintended pandemic pregnancy. Contraceptive use remains a crucial preventive strategy to averting unintended pregnancy, particularly for married young women.

For peer review only

Strengths and limitations of the study

- The COVID-19 pandemic raised concerns for unintended pregnancy for adolescent girls and young women (AGYW), however, until now, data on this distal outcome had not been available due to time lag.
- To our knowledge this is the first study to longitudinally examine unintended pregnancy incurred during the COVID-19 pandemic among AGYW in sub-Saharan Africa; limitations of retrospective unintended pregnancy assessment are well established, making prospective cohort data uniquely valuable to address this topic.
- The assessment of unintended pandemic pregnancy, while our best estimate, may be subject to measurement error given that data collection was not continually ongoing throughout the pandemic.
- Data were collected immediately prior to the pandemic and at two points after the implementation of COVID-19 restrictions in Nairobi—as Kenya continues to experience COVID-19 infection waves and reinstates mobility restrictions to curb infection spread, ongoing data collection is needed to understand impacts.
- Nairobi is a major urban center and results should not be generalized to all parts of Kenya, where AGYW may face different challenges surrounding sexual and reproductive health.

For peer review only

For peer review only - http://bmjopen.bmj.com/site/about/guidelines.xhtml

Introduction

Adolescent pregnancy is a human rights concern with gendered repercussions that disproportionately impact adolescent girls and young women (AGYW) (1,2). Both intended and unintended pregnancies can alter AGYW's life trajectories, including by decreasing their ability to meet educational, business/financial, and family goals (1). Further, early pregnancy can have negative health effects—adolescents are at risk for increased birth complications, including obstructed labor, fistula, premature delivery, low birth weight, pre-eclampsia/eclampsia, and maternal and perinatal mortality, compared to their older counterparts (1,3). While complete adolescent pregnancy data are difficult to obtain, largely due to underreporting and stigmatization of unintended pregnancies that end in abortions, the evidence is clear that the majority of the global adolescent pregnancy burden is concentrated within sub-Saharan Africa, where adolescent pregnancy rates in most countries exceed 100 per 1,000 women aged 15-19 (4).

Motivations and intentions surrounding adolescent pregnancy are complex and require a socioecological lens (5). Pregnancy intendedness varies substantially by setting and in many contexts, cultural and familial norms may promote early childbearing in lieu of economic and educational attainment (2). Factors linked to adolescent pregnancy in sub-Saharan Africa include low education, lack of familial education, poverty, and limited communication/knowledge surrounding sexual and reproductive health (SRH) (6,7). Ecological data further correlate adolescent pregnancy with high gender inequality at the national level (8). While rural adolescents are generally at heightened risk of pregnancy, largely due to decreased access to SRH services (6,7), urban adolescents have long been a target group for SRH programs given the population's growing size and unique needs (9). Many AGYW migrate to cities to seek out financial opportunities, but instead are met with stigma and shame when seeking healthcare services (10,11); these barriers may be more pronounced for unmarried youth given social norms dictating sex as a means for procreation and stigmatization of sexual activity outside of marriage, and in turn, increase unmet SRH needs (12).

When the COVID-19 outbreak was declared a pandemic by the World Health Organization (WHO) in March 2020, the SRH community was concerned about the negative impact mitigation measures would have on women's SRH, and particularly the SRH of AGYW (13–15). Early COVID-19 projections estimated that 15 million additional unintended pregnancies could occur over one year if COVID-related service disruptions affected 10% of women in need of SRH services in low- and middle-income countries (16). Unintended pregnancy is a distal outcome and requires time and prospective data to accurately assess; thus far, no studies have corroborated these pregnancy projections (17,18). In lieu of unintended pregnancy data, contraceptive use is the most widely used proxy for unintended pregnancy—population-level data from four sub-Saharan African contexts indicate that contraceptive use among women in need increased across settings, contrary to warnings of unintended pregnancy increases (19). While early trends are largely promising, some groups may be at increased risk of unintended pregnancy, namely adolescents and nulliparous women, due to increases in contraceptive need and reductions in contraceptive use during this period (19). Another study utilizing facility data to assess facility admissions and antenatal, delivery, and immunization services reported slight reductions in services (20). Adolescents may further face difficulties procuring contraception during the COVID-19 pandemic given limited household bargaining power and school closures (21,22). Recent causal-comparative research from Western Kenya elucidates increased school dropout and pregnancy among specific to secondary school girls during COVID-19 compared to a pre-COVID-19 cohort (23).

While multiple studies have examined youth contraceptive use and/or sexual activity in light of COVID-19 (17,18,21,22), no study to date has utilized prospective data to examine unintended pregnancy during the COVID-19 pandemic in sub-Saharan Africa for AGYW. Among urban youth in Nairobi, Kenya, this study aimed to 1) characterize fertility and contraceptive use dynamics by gender; 2) estimate pregnancy prevalence for AGYW over the course of the pandemic; and 3) assess factors associated with unintended pandemic pregnancy for young women.

Methods

Study Setting

This study takes place in urban Nairobi, Kenya, where adolescent pregnancy is a country priority (24)—per 2014 Demographic and Health Survey estimates, one-fifth of Kenyan AGYW ages 15-19 were pregnant or already mothers, and approximately half of unmarried, sexually active AGYW reported using a modern method of contraception (25). The COVID-19 pandemic elevated mitigation of adolescent pregnancy within national policies. The first case of COVID-19 in Kenya was detected on March 13, 2020 (26), with school closures, national lockdown, and mandatory curfew immediately following (27). By late 2020 restrictions were eased; however, in summer 2021, caseloads began rising again, prompting additional closures (27). These restrictions, while essential to curbing the spread of COVID-19, were hypothesized to decrease access to essential health services, including SRH services.

For peer review only - http://bmjopen.bmj.com/site/about/guidelines.xhtml

Study Overview

This longitudinal study draws on data collected from the Nairobi Youth Respondent Driven Sampling Survey (YRDSS), a cohort of adolescents and young adults in Nairobi collected at three time points prior to and during the COVID-19 pandemic: June-August 2019 [pre-pandemic], August-October 2020 [12-month follow-up], and April-May 2021 [18-month follow-up].

Briefly, the YRDSS began recruitment in June-August 2019 using respondent-driven sampling (RDS), a chain-based recruitment method that begins with purposefully selected seeds, followed by monitored peer-to-peer coupon distribution (28). At the time of recruitment, eligible youth were aged 15-24 years, unmarried, and residing in Nairobi for at least one year. The 2019 pre-pandemic survey round recruited 1,357 participants; of whom 95% (1,293/1,357) consented for recontact and provided contact information. This cohort was recontacted and surveyed in August-October 2020 (12-month follow-up; n=1,217; 94% retention], and in April-May 2021 (18-month follow-up; n=1,177; 97% retention).

Ethical approval was obtained from the Ethics Review Committee at Kenyatta National Hospital/University of Nairobi (P294/04/2019) and the Institutional Review Board at Johns Hopkins Bloomberg School of Public Health (00009496). Detailed study procedures are outlined elsewhere (29).

Data Collection Procedures

The pre-pandemic [2019] survey was specific to reproductive health needs and family planning behaviors for adolescents and young adults. Data collection occurred in-person with surveys self-administered by participants or administered by trained resident enumerators in case of comprehension or technological difficulty. Data was entered onto tablets and uploaded to a cloud server using Open Data Kit (ODK) software. Surveys were conducted in English or Swahili, based on participant preference.

Given the onset of the COVID-19 pandemic, follow-up surveys at 12-months [2020] and 18-months [2021] were designed specifically to examine the gendered impacts of COVID-19 on youth economic, health, and social experiences, including pandemic impacts on youth contraceptive use and unintended pregnancy. In adherence to COVID-19 restrictions, interviews at these timepoints were conducted by the same resident enumerators via remote phone interviews, with responses recorded using the ODK software.

Measures

Outcomes. Primary outcomes comprised fertility and contraceptive use for both genders, as well as pregnancy for young women.

Prospective fertility intentions were assessed per gender at each of the three time points. Specifically, each participant was asked "How long would you like to wait from now before the birth of a child?" with responses comprising "x months", "x years", "would like to get pregnant soon/now", and "can't get pregnant" (for women) or "can't cause pregnancy" (for men), and "do not want children." For trend analysis, intentions were categorized as "currently pregnant"; "<1 year"; "1-2 years"; ">2 years/other."

Current contraceptive use was assessed per gender at each time point utilizing standard items (30). Contraceptive users were asked current method, and if users reported more than one current method, they were then asked to identify the method they used "mainly" or "most of the time". Responses were split into method effectiveness categories for trend analysis (1-long-acting reversible contraception (LARC): intrauterine device (IUD) and implant; 2-short-acting: injectable and pills; 3-coital-dependent: emergency contraception, male condoms, and female condoms; 4-other/traditional: rhythm, withdrawal, and herbal pill method).

Period and current pregnancy were assessed per time point for young women. At pre-pandemic baseline, young women were asked about ever pregnancy, whereas at 12-month and 18-month follow-up, past 12-month and past 6-month pregnancy were assessed to capture between-survey outcomes. For all young women with a period pregnancy, current pregnancy assessed via single item, "Are you currently pregnant?" A dichotomous variable was then created for the primary outcome of interest, unintended pandemic pregnancy, defined as having a current pregnancy or past 6-month pregnancy at 18-month follow-up survey and wanting to wait more than one year for a pregnancy at last survey round (2020).

For peer review only - http://bmjopen.bmj.com/site/about/guidelines.xhtml

Exposures for unintended pandemic pregnancy: All risk factors for unintended pandemic pregnancy were assessed at 12-month follow-up (2020) survey using standard measures (29,30), and include age (15-19; 20-24); highest level of education completed (no school/primary/post-primary; secondary or higher); subjective household wealth tertile (low; middle; high); employment (someone else vs. self); increased time with partner since COVID-19 restrictions (yes/no); ability to meet basic needs (yes/no); and contraceptive use at 2020 survey. Exposures were dichotomized based on underlying distributions to maximize power for multivariable analyses.

Analytic Sample

Within timepoint analyses were restricted to participants with completed survey data for that round (n=605 young men, n=610 young women at pre-pandemic and 12-month follow-up; n=586 young men, n=591 young women at 18-month follow-up). Trend and prospective analyses were restricted to those with complete data at all three timepoints (n=586 young men, n=589 young women); for contraceptive dynamics, analyses were additionally restricted to those with a need for contraception (i.e., sexually active, not pregnant (if female), and not wanting a child within the next year) at both the time of that survey and the prior survey round. A complete case approach was adopted given <1% missing outcome data (n=2 missing unintended pandemic pregnancy); sample size floats to accommodate small amounts of missing covariate data (<1%).

Statistical Analysis

Descriptive statistics examined characteristics of youth at pre-pandemic, by gender, with design-based F-statistics classifying between-gender differences. Sankey diagrams depicted dynamics in fertility intentions and contraceptive use across the three survey rounds, by gender. Among youth who discontinued their method at 12-month or 18-month follow-up, main reason for discontinuation was examined, per gender and time point. For each round, period and current pregnancy prevalence were calculated for all young women, and among adolescent girls (<18-years-old) and those wishing to avert a pregnancy at last round (i.e., did not want a child within the next year). Lastly, bivariate and multivariable logistic regression examined factors at 12-month follow-up (2020) associated with unintended pandemic pregnancy among young women via glm models with family binomial and link logistic to account for weighing and clustering. Factors with p-values <0.1 from bivariate models were included within multivariable models, withstanding increased time with partner, omitted due to collinearity.

All analyses were conducted using Stata 16 (College Station, TX) with statistical significance set a priori at p<0.05. Sampling weights accommodate the respondent driven sampling study design using RDS-II (Volz-Heckathorn) weights, post-estimation adjustment based on 2014 Kenya Demographic and Health Survey population data (age, sex, education levels), and loss-to-follow-up. All estimates are weighted; statistical testing accounts for clustering among participants recruited by the same recruiter at baseline.

Patient and Public Involvement

This community-engaged study sought public and end-user input at all phases. During the formative research stage prior to the 2019 cohort recruitment, input from community-based, youth-serving organizations informed the study recruitment strategy for feasibility, survey measures and constructs to ensure relevance, and study logistics to maximize participant comfort and confidentiality. All recruitment and procedures were conducted by trained resident enumerators selected from underlying communities, and who provided inputs on measures for clarity and aided in results interpretation. Findings were disseminated in November 2020 and again in September 2021 with stakeholders spanning policy sector, government representatives, elders/faith leaders, community-based organizations, and youth leaders from the study communities.

Results

Characteristics of youth at pre-pandemic, by gender, are outlined in Table 1. The majority of both young men and young women were aged 20-24 (60.7% and 57.2%, respectively) and had completed at least secondary education (62.5% and 57.2%, respectively). While roughly three of four youth had ever had sex, higher proportions of young men reported current involvement in a romantic relationship (young men: 66.5%; young women: 54.5%; p=0.04). Approximately one in ten young men had a partner who had ever been pregnant and 8.0% of young women reported their own pregnancy experience. Future fertility intentions differed by gender, with higher proportions of young men reporting wanting a child soon/now (2.7% young men vs. 0.2% young women) or within the next two years (15.8% young men vs. 11.0% young women; p=0.05). Contraceptive use, method effectiveness, and main current contraceptive method differed significantly by gender. Approximately three-quarters of young men reported current

For peer review only - http://bmjopen.bmj.com/site/about/guidelines.xhtml

contraceptive use (75.2%), compared to half of young women (54.7%; p<0.001). The majority (91.2%) of young men reported male condom use as their main contraceptive method, whereas young women utilized a range of methods (p<0.001).

Table 1. Characteristics of youth at pre-pandemic (2019; n=1,215), weighted

	Young Men (n=605)	Young Women (n=610)	p-value[§]
	n (column %)		
Demographic Characteristics			
Age			0.38
15-19	238 (39.3)	261 (42.8)	
20-24	367 (60.7)	349 (57.2)	
Highest level of education completed			0.62
Post-primary/primary or no school	197 (32.6)	211 (34.7)	
Secondary or higher	408 (67.4)	399 (65.4)	
Relationship and Reproductive Characteristics			
Ever had sex	436 (72.0)	441 (72.3)	0.94
Currently in a romantic/sexual relationship	402 (66.5)	332 (54.5)	**0.04**
Ever pregnant/partner has ever been pregnant	62 (10.2)	49 (8.0)	0.35
Future fertility intentions			**0.05**
Soon/now	16.3 (2.7)	1 (0.2)	
Within the next two years	96 (15.8)	67 (11.0)	
More than two years	410 (67.7)	446 (73.2)	
Does not want children or cannot have children	7 (1.1)	8 (1.4)	
Don't know	76 (12.6)	87 (14.2)	
Contraceptive Characteristics			
Contraceptive use	323 (75.2)	249 (54.7)	**<0.001**
Method effectiveness[±]			**<0.001**
Long-acting reversible contraception	3 (1.0)	48 (19.5)	
Short-acting	2 (0.5)	55 (22.5)	
Barrier or hormonal coital-dependent	286 (93.6)	114 (46.5)	
Other/traditional	15 (4.9)	28 (11.4)	
Main Contraceptive Method[±]			**<0.001**
Implant	3 (1.0)	48 (19.1)	
Coil/IUD	0 (0.0)	7 (2.7)	
Injectable	2 (0.5)	43 (17.0)	
Oral contraceptive pills	0 (0.0)	12 (5.0)	
Emergency contraception	6 (1.9)	24 (9.7)	
Male condom	281 (91.2)	85 (34.0)	
Female condom	0 (0.0)	5 (1.8)	
Cycle beads/Standard Days Method/rhythm	7 (2.4)	12 (4.6)	
Withdrawal	8 (2.5)	10 (3.9)	
Herbal Pill Method	1 (0.5)	5 (2.1)	
Place Obtained Main Contraceptive Method[±]			0.28
Hospital	26 (9.1)	34 (15.1)	
Health Center	89 (30.1)	69 (30.9)	
Clinic/Other Health Provider	26 (9.1)	31 (13.7)	
Pharmacy	91 (31.7)	62 (27.6)	
Other	55 (19.1)	28 (12.7)	

For peer review only - http://bmjopen.bmj.com/site/about/guidelines.xhtml

§p-value from design-based F-statistic; boldface indicates $p<0.05$
±Among users of contraception
Method effectiveness: LARC=IUD, implant; short-acting=injectable, pills; coital-dependent=emergency contraception, male condoms, female condoms; other/traditional=rhythm, withdrawal, herbal pill method
Bolding indicates $p<0.05$

Throughout the pandemic, young women's fertility intentions remained relatively stable (Figure 1). Between pre-pandemic and 12-month follow up [2020], largest shifts in fertility intention were seen among women who wanted to wait more than two years at pre-pandemic (88.6%; n=515/591) and then said they wanted a child in 1-2 years at 2020 (11.4%; n=80/591); this trend was similarly observed between 12-month and 18-month follow-up, wherein 9.9% of all women (n=65/591) shifted from the greater than 2 years category to 1-2 years. The second largest shift was seen among young women who wanted to wait more than two years at pre-pandemic, but then reported pregnancy at 12-month [2020] follow-up (4.2%; n=17/591). From 12-month to 18-month follow-up, a corresponding shift from pregnancy to wanting to wait more than two years was reported (4.8%; n=20/591).

Trends in fertility intentions for young men (Figure 1) were similar; however, between 12-month [2020] and 18-month [2021] follow-up, large portions of young men also sought to delay childbearing, with 6.8% changing from wanting a child within 1-2 years to wanting to wait two or more years (n=35/586).

[Figure 1 here]

Contraceptive dynamics throughout the pandemic showed substantial variation by gender (Figure 2). Specifically, young men largely migrated between coital-dependent method use and non-use at both timepoints (including both adoption and discontinuation). Conversely, many young women began as non-users and adopted coital-dependent (33.5%; n=67/192) or short-acting methods (13.3%; n=30/192) at 12-month follow-up [2020]. While some coital-dependent users at 12-month follow-up [2020] discontinued by 18-month follow-up (26.2%; n=28/113) [2021], some non-users also continued to take up coital-dependent (15.2%; n=25/120), short-acting (5.0%; n=6/120), and LARC methods (11.4%; 12/120).

[Figure 2 here]

At both 2020 and 2021 surveys and across genders, the primary reason for contraceptive discontinuation centered around partners, including fear of cheating, partner opposed, partner away, infrequent sex, not married (Young women_{2020}: 64%; Young women_{2021}: 62%; Young men_{2020}: 66%; Young men_{2021} 76%; Figure 3). Additionally, approximately one-fifth of both young women (21%) and young men (22%) at 2020 survey stated discontinuation for pregnancy-related reasons, namely wanting to become pregnant. COVID-related, access-related, and personal concerns were less common.

[Figure 3 here]

Following trends in fertility intentions and contraceptive use, prevalence of pregnancy differed across survey rounds (Table 2). Period prevalence ranged from 31.4% ever pregnant at pre-pandemic (2019) to 6.8% pregnant in the past 6-months at 18-month follow-up (2021). Current pregnancy was highest at the 12-month follow-up survey (2020) at 4.8%, whereas it hovered at approximately two percent at the 2019 and 2021 survey rounds. Unintended pandemic pregnancy (i.e., current pregnancy or past 6-month pregnancy at the 18-month follow-up survey with no intention to get pregnant indicated at the previous survey round) was 6.1%, with prevalence approximately half for girls less than age 18 (3.3%).

For peer review only - http://bmjopen.bmj.com/site/about/guidelines.xhtml

Table 2. Prevalence of pregnancy across survey rounds, weighted

	All AGYW	Adolescent girls (<18 years old at baseline)	Among AGYW wishing to avert a pregnancy at last round§
	% (n)		
Period prevalence			
Pre-pandemic (2019) ever pregnant	31.4 (191)	6.2 (5)	--
12-month follow-up (2020) past 12-month pregnancy	12.5 (76)	1.2 (1)	11.2 (65)
18-month follow-up (2021) past 6-month pregnancy	6.8 (40)	3.3 (1)	6.6 (36)
Current pregnancy			
Pre-pandemic (2019)	1.8 (11)	0.0 (0)	--
12-month follow-up (2020)	4.8 (29)	0.0 (0)	4.6 (27)
18-month follow-up (2021)	2.0 (12)	3.3 (1)	1.6 (9)
Unintended pandemic pregnancy (current pregnancy or past-6-month pregnancy at 18-month follow-up, with no intention to get pregnant at last round (2020)	6.1 (36)	3.3 (1)	--

§Wished to wait for pregnancy >1 year at pre-pandemic (for 12-month follow-up assessment) and >6 months at 12-month follow-up (for 18-month follow-up assessment)

Table 3 presents bivariate and multivariable factors at 12-month follow-up (2020) associated with unintended pandemic pregnancy. Recent marriage (at 2020 survey) was associated with a greater than four-fold increase in unintended pandemic pregnancy in both bivariate (OR=4.89; 95% CI=1.93-12.37; p<0.001) and multivariable models (aOR=4.16; 95% CI=1.65-10.51; p=0.006). Within the bivariate model, increased time with partner since COVID-19 restrictions was also associated with increased risk of unintended pandemic pregnancy (OR=3.69; 95% CI=1.61-8.46; p=0.002), however, was excluded from multivariable models due to collinearity with marital status. Contraceptive use at 2020 survey contributed to an 80% reduction in unintended pandemic pregnancy (aOR=0.21; 95% CI=0.09-0.50; p<0.001). Additionally, within both bivariate models, young women with an inability to meet basic needs displayed a borderline protective effect against unintended pandemic pregnancy (OR=0.47; 95% CI=0.21-1.05; p=0.07); however, this association attenuated when adjusted.

Table 3. Factors at 12-month Follow-up (2020) Associated with Unintended Pandemic Pregnancy for Young Women (n=589)

	Unintended Pandemic Pregnancy Row %	OR (95% CI)	aOR (95% CI)
Factors Assessed at 12-month follow-up (2020)			
Age			
15-19	6.5	Ref	--
20-24	5.9	0.90 (0.37, 2.19)	--
Highest level of education completed			
Post-primary/primary or no school	6.9	Ref	--
Secondary or higher	5.7	0.82 (0.32, 2.08)	--
Household wealth			
Low	6.4	Ref	--
Middle	5.6	0.87 (0.33, 2.34)	--
High	6.2	0.96 (0.34, 2.70)	--
Married			
No	4.5	Ref	ref
Yes	18.9	**4.89 (1.93, 12.37)** ***	**4.16 (1.65, 10.51)** **
Employed			
Student, caregiver, unemployed	5.1	Ref	--
Employed formally or informally	7.2	1.45 (0.60, 3.49)	--
Prime earner in household			

For peer review only - http://bmjopen.bmj.com/site/about/guidelines.xhtml

Someone else	5.9	Ref	--
Self	7.4	1.28 (0.46, 3.57)	--
Increased time with partner since COVID-19 restrictions			
No	3.8	Ref	--
Yes	12.8	**3.69 (1.61, 8.46)** **	--
Ability to meet basic needs			
No	4.1	0.47 (0.21, 1.05) œ	0.54 (0.23, 1.29) œ
Yes	8.4	Ref	ref
Contraceptive use			
No	14.4	Ref	ref
Yes	3.7	**0.23 (0.10, 0.53)** ***	**0.21 (0.09, 0.50)** ***

Increased time with partner omitted from multivariable model due to collinearity
Bolding indicates p<0.05

Discussion

Within a narrative that has largely focused on shifting fertility intentions and contraceptive use amidst the COVID-19 pandemic (16,19,31), these longitudinal results counter those for adult populations (17,18) and indicate that fears of unintended pregnancy for AGYW were indeed warranted. AGYW in Nairobi, Kenya did not use effective methods of contraception and in turn, experienced unintended pregnancy during the time of highest COVID-19 mitigation measures (2020 survey). Specifically, current pregnancy more than doubled at the 2020 survey wave (4.8%) versus pre-pandemic (1.8%) or 2021 (2.0%) surveys, and the majority of pregnancies at 2020 survey (93%) were unintended. Counter to initial hypotheses, education and economic insecurity did not pose significant threats to unintended pandemic pregnancy—instead, risk among AGYW in Nairobi largely centered on newly initiated marriage.

Notably, recent marriage was the only risk factor associated with increased odds of unintended pandemic pregnancy within the multivariable models, where young women who recently married had a four-fold increase of unintended pandemic pregnancy, as compared to those who remained unmarried (p<0.001). Such findings bolster recent results from national cohorts of adult women predicting increases in unintended pregnancy for nulliparous and young women in Lagos, Nigeria (19); the aforementioned study, however, did not highlight potential increases in unintended pregnancy for young women within Kenya (19). Utilizing longitudinal data from an existing cohort of this hard-to-reach population, our findings elucidate that it may not be age nor parity driving risk for AGYW, but rather power dynamics within a newly formed marriage. Pressures for newly married women may increase risk of unintended pregnancy, both during the COVID-19 pandemic and after, and require further research to understand relationship dynamics for newly married couples. While partner pressures are most prominent, concurrent pressures from family and community members should not be downplayed (32,33), and a socioecological framework should be adopted in future research to fully understand the risks of new marriage for AGYW's SRH. While programs to increase age at marriage and keep girls in school are vital to ensure AGYW's autonomy in the decision to marry and their future livelihoods, interventions to increase relationship quality and help young couples navigate the complexities of this new life stage must be simultaneously prioritized in order for young couples to prosper both individually and jointly.

Shifts in fertility intentions throughout the pandemic largely followed expected patterns and timelines (i.e., women who wanted to wait more than two years at pre-pandemic stated that they then wanted a child within 1-2 years one year later). Some AGYW (4.2%; n=17), however, did report wanting to wait more than two years at pre-pandemic survey but then had an unintended pregnancy at 2020 survey—at 2021 survey, these young women again wanted to wait more than two years for their next pregnancy. While young men's fertility intentions followed similar patterns, between 12-month [2020] and 18-month [2021] follow-up, 6.8% shifted from wanting a child within 1-2 years to wanting to wait two or more years—this shift in intentions could be in part due to the continued economic uncertainty and instability imposed by the COVID-19 pandemic. Results corroborate borderline significant results in multivariable models indicating that AGYW with an inability to meet their basic needs had a 50% reduction in unintended pandemic pregnancy. Ultimately, these results speak to the resilience of young people, as well as their initiative to prepare and financially plan for a pregnancy. Continued investment in youth economic empowerment can ensure young people are able to meet both their SRH and financial goals.

For peer review only

Moreover, results affirm the effectiveness of primary prevention for unintended pregnancy via contraceptive use. Contraceptive use at previous survey round was the only factor protective against unintended pandemic pregnancy—AGYW who used contraception, regardless of method, had an 80% reduction in unintended pandemic pregnancy, compared to those who were not using contraception at last survey round. Youth must have continued access to non-stigmatizing, youth-friendly, and low-cost SRH services. For AGYW who experience unintended pregnancies, during the COVID-19 pandemic and otherwise, access to postpartum contraception is imperative to deter against rapid repeat pregnancy, while ensuring that timing of future pregnancies aligns with a woman's reproductive intentions.

While early research projected contraceptive use decreases due to COVID-related reasons (16), urban AGYW in Nairobi did not cite inability to go to the facility due to COVID-19 restrictions as their primary reason for discontinuation. At both 2020 and 2021 and across genders, the primary reason for contraceptive discontinuation centered around partners (fear of cheating, partner opposed, partner away, infrequent sex, not married). Notably, the included survey item for COVID-related reasons only measured access challenges via inability to visit facilities due to restrictions. Moreover, respondents were only instructed to select their *primary* reason for discontinuation, though multiple reasons could have been experienced. Recent COVID-19 research has reported negative impacts of COVID-19 on youth relationships (21,22,34), including dissolution of partnerships, increased economic dependency, intimate partner violence, and accelerated marriage timelines; altogether, the current research base describes the difficulty of disentangling reasons for discontinuation, with partnership dynamics potentially serving as a proxy for more distal COVID-related reasons. Partnership and COVID-factors should continue to concurrently be measured in survey research, with further longitudinal research needed to disentangle their full and partial effects on SRH outcomes.

Results should be interpreted considering study limitations. Foremost, data were collected immediately prior to the pandemic and at two points after the implementation of COVID-19 restrictions in Nairobi—as Kenya continues to experience COVID-19 infection waves and reinstates mobility restrictions to curb infection spread, ongoing data collection is needed to understand impacts on AGYW's SRH and well-being. Moreover, the assessment of unintended pandemic pregnancy, while our best estimate, may be subject to measurement error given that data collection was not continually ongoing throughout the pandemic. Namely, some AGYW who were pregnant at the 2020 survey round may have indeed experienced an unintended pandemic pregnancy, although we utilized the 18-month (2021) survey for more accurate estimates. We further recognize that fertility intentions are dynamic; while we utilized prospective intention measures from the most recent previous time point to minimize recall biases associated with intention measurement, we recognize that fertility intentions could shift throughout our short follow-up window. Period pregnancy prevalence estimates utilize different referent timepoints and therefore are not comparable; as such, significance testing between times was not utilized and these data should be interpreted as purely descriptive. Nairobi is a major urban center and results should not be generalized to all parts of Kenya, where AGYW may face different challenges surrounding SRH.

As of February 2023, Kenya has experienced seven COVID-19 infection waves, and the health and economic impacts of these waves may not yet to be fully uncovered; recent notable policy shifts, however, signal positive steps for youth in the country (35). Growing momentum seeks to advance women's safety and reproductive rights, including via affirming access to abortion services, and advocating for the elimination of gender-based violence as a national priority. These prospective data, however, highlight that continued policies are needed to affirm reproductive rights and access to quality contraceptive and SRH care for adolescents and young adults. Foremost, continuous access to quality health education, youth-friendly contraceptive services, and universal health care more broadly is imperative to protect the reproductive autonomy of both young men and young women. Further evidence-informed policy efforts should center on dismantling fertility pressures within the community and partner dyad, particularly for AGYW transitioning from dating to marital relationships and still seeking to avert pregnancy. Additional data collection in this arena can aid in monitoring fertility pressures, contraceptive access and use, and quality of care to better understand the continued SRH needs of this vulnerable group. Such youth-friendly policies are crucial for advancing adolescent and young adult SRH both in times of global public health crises and in times of relative stability.

For peer review only - http://bmjopen.bmj.com/site/about/guidelines.xhtml

Funding: This work was supported, in whole, by the Bill & Melinda Gates Foundation [010481]. Under the grant conditions of the Foundation, a Creative Commons Attribution 4.0 Generic License has already been assigned to the Author Accepted Manuscript version that might arise from this submission. The funders had no role in the study design, collection, analysis, and interpretation of data, in writing of the report, or in the decision to submit for publication.

Contributors: Shannon N. Wood contributed to study design, literature search, data analysis, data interpretation, and writing. Meagan E. Byrne contributed to study design, literature search, data analysis, figures, data interpretation, and writing. Mary Thiongo contributed to study design, data interpretation, and writing. Bianca Devoto contributed to study design, data interpretation, and writing. Grace Wamue-Ngare contributed to data interpretation and writing. Michele R. Decker contributed to study design, data interpretation, and writing. Peter Gichangi contributed to study design, data collection, data interpretation, and writing. All authors read and approved the final version of this manuscript.

Ethics Approval: Ethical approval was obtained from the Ethics Review Committee at Kenyatta National Hospital/University of Nairobi (P294/04/2019) and the Institutional Review Board at Johns Hopkins Bloomberg School of Public Health (00009496).

Declaration of interests: No conflicts of interest to disclose.

Acknowledgements: We acknowledge Dr. Caroline Moreau for her assistance in defining our analytic sample and outcome variables.

Data Sharing: Data are available upon request from www.pmadata.org.

For peer review only

References

1. UNFPA. Motherhood in Childhood [Internet]. The State of the World Population 2013. 2013. Available from: http://www.unfpa.org/sites/default/files/pub-pdf/EN-SWOP2013.pdf
2. Chandra-Mouli V, Camacho AV, Michaud PA. WHO guidelines on preventing early pregnancy and poor reproductive outcomes among adolescents in developing countries. J Adolesc Heal. 2013;52(5):517–22.
3. Grønvik T, Sandøy IF. Complications associated with adolescent childbearing in Sub-Saharan Africa: A systematic literature review and meta-analysis. PLoS One. 2018;13(9):1–21.
4. Sedgh G, Finer LB, Bankole A, Eilers MA, Singh S. Adolescent Pregnancy, Birth, and Abortion Rates Across Countries: Levels and Recent Trends. J Adolesc Heal [Internet]. 2015;56(2):223–30. Available from: http://dx.doi.org/10.1016/j.jadohealth.2014.09.007
5. Blum RW, Bastos FI, Kabiru CW, Le LC. Adolescent health in the 21st century. Lancet. 2012;379(9826):1567–8.
6. Kassa GM, Arowojolu AO, Odukogbe AA, Yalew AW. Prevalence and determinants of adolescent pregnancy in Africa: A systematic review and Meta-analysis 11 Medical and Health Sciences 1117 Public Health and Health Services. Reprod Health. 2018;15(1):1–17.
7. Yakubu I, Salisu WJ. Determinants of adolescent pregnancy in sub-Saharan Africa: A systematic review. Reprod Health. 2018;15(1).
8. Decker MR, Kalamar A, Tunçalp Ö, Hindin MJ. Early adolescent childbearing in low- and middle-income countries: Associations with income inequity, human development and gender equality. Health Policy Plan. 2017;32(2):277–82.
9. Melesse DY, Mutua MK, Choudhury A, Wado YD, Faye CM, Neal S, et al. Adolescent sexual and reproductive health in sub-Saharan Africa: Who is left behind? BMJ Glob Heal. 2020;5(e002231).
10. Stidham Hall K, Manu A, Morhe E, Dalton V, Challa S, Loll D, et al. Bad girl and unmet family planning need among Sub-Saharan African adolescents: the role of sexual and reproductive health stigma. Qual Res Med Healthc. 2018;2(1):55–64.
11. Wood K, Jewkes R. Blood Blockages and Scolding Nurses: Barriers to Adolescent Contraceptive Use in South Africa. Reprod Health Matters. 2006;14(27):109–18.
12. Chandra-Mouli V, McCarraher DR, Phillips SJ, Williamson NE, Hainsworth G. Contraception for adolescents in low and middle income countries: Needs, barriers, and access. Reprod Health. 2014;11(1):1–8.
13. Hussein J. COVID-19: What implications for sexual and reproductive health and rights globally? Sex Reprod Heal Matters. 2020;28(1).
14. Thomas AB, Yaya S. Looking ahead in the COVID‑19 pandemic : emerging lessons learned for sexual and reproductive health services in low‑ and middle‑ income countries. Reprod Health. 2021;4:1–8.
15. Marie Stopes International. COVID-19 threatens women's health [Internet]. 2020. Available from: https://mariestopes-us.org/2020/covid-19-threatens-womens-health/
16. Riley T, Sully E, Ahmed Z, Biddlecom A. Estimates of the Potential Impact of the COVID-19 Pandemic on Sexual and Reproductive Health In Low- and Middle-Income Countries. Int Perspect Sex Reprod Health. 2020;46:73–6.
17. Moreau C, Karp C, Wood S, Williams K, Olaolorun FM, Akilimali P, et al. Trends in fertility intentions and contraceptive practices in the context of COVID-19 in sub-Saharan Africa: insights from four national and regional population-based cohorts. BMJ Open. 2023;13(1):e062385.
18. Backhaus A. Pregnancies and contraceptive use in four African countries during the COVID-19 pandemic. Vienna Yearb Popul Res. 2022;20:1–18.
19. Wood SN, Karp C, OlaOlorun F, Akilimali P, Guiella G, Gichangi P, et al. Trends in the need for and use of contraception before and during COVID-19 in four sub-Saharan African geographies: results from population-based national or regional cohort surveys. Lancet Glob Heal. 2021;9:e793-801.
20. Amouzou A, Maïga A, Faye CM, Chakwera S, Melesse DY, Mutua MK, et al. Health service utilisation during the COVID-19 pandemic in sub-Saharan Africa in 2020: A multicountry empirical assessment with a focus on maternal, newborn and child health services. BMJ Glob Heal. 2022;7(5).
21. Karp C, Moreau C, Sheehy G, Anjur-Dietrich S, Mbushi F, Muluve E, et al. Youth Relationships in the Era of COVID-19: A Mixed-Methods Study Among Adolescent Girls and Young Women in Kenya. J Adolesc Heal. 2021;69(5):754–61.
22. Decker MR, Wood SN, Thiongo M, Byrne ME, Devoto B, Morgan R, et al. Gendered health, economic, social and safety impact of COVID-19 on adolescents and young adults in Nairobi, Kenya. PLoS One.

For peer review only - http://bmjopen.bmj.com/site/about/guidelines.xhtml

2021;16(11):e0259583.
23. Zulaika G, Bulbarelli M, Nyothach E, Van Eijk A, Mason L, Fwaya E, et al. Impact of COVID-19 lockdowns on adolescent pregnancy and school dropout among secondary schoolgirls in Kenya. BMJ Glob Heal. 2022;7(1):1–9.
24. National Council for Population and Development. Draft Action Plan for Addressing Adolescent Health and Teenage Pregnancy in Kenya [Internet]. 2021. Available from: https://ncpd.go.ke/wp-content/uploads/2021/10/Draft-Action-Plan-for-Addressing-Adolescent-Health-and-Teenage-Pregnancy-in-Kenya.pdf
25. DHS. Kenya 2014 Demographic and Health Survey.
26. Quaife M, van Zandvoort K, Gimma A, Shah K, McCreesh N, Prem K, et al. The impact of COVID-19 control measures on social contacts and transmission in Kenyan informal settlements. BMC Med. 2020;18(316):1–11.
27. Yusuf M. Kenya Sees New Surge of Coronavirus After Easing Restrictions [Internet]. Voices of America. 2020 [cited 2020 Oct 19]. Available from: https://www.voanews.com/a/covid-19-pandemic_kenya-sees-new-surge-coronavirus-after-easing-restrictions/6197319.html
28. Magnani R, Sabin K, Saidel T, Heckathorn D. Review of sampling hard-to-reach and hidden populations for HIV surveillance. AIDS. 2005;19(Supplement 2):67–72.
29. International Centre for Reproductive Health-Kenya (ICRHK) & PMA Agile. Nairobi Youth Respondent-Driven Sampling Survey: Final Report. Baltimore, Maryland; 2020.
30. Performance Monitoring for Action (PMA). Performance Monitoring for Action Survey Methodology [Internet]. 2021. Available from: https://www.pmadata.org/data/survey-methodology
31. Karp C, Wood SN, Guiella G, Gichangi P, Bell SO, Anglewicz P, et al. Contraceptive dynamics during COVID-19 in sub- -Saharan Africa: longitudinal evidence from Burkina Faso and Kenya. BMJ Sex Reprod Heal. 2021;0:1–9.
32. Karp C, Wood SN, Hadiza G, Kibira SPS, Makumbi F, Omoluabi E, et al. "I am the master key that opens and locks": Presentation and application of a conceptual framework for women's and girls' empowerment in reproductive health. Soc Sci Med. 2020;258(113086).
33. Wood SN, Kennedy SR, Akumu I, Tallam C, Asira B, Hameeduddin Z, et al. Correlates and contributors of reproductive coercion across the socioecological framework among intimate partner violence survivors in Nairobi, Kenya. J Fam Violence. 2022;
34. Decker MR, Bevilacqua K, Wood SN, Ngare GW, Thiongo M, Byrne ME, et al. Gender-based violence during COVID-19 among adolescent girls and young women in Nairobi, Kenya: a mixed-methods prospective study over 18 months. BMJ Glob Heal. 2022;7(2):1–14.
35. Our World in Data. Policy Responses to the Coronavirus Pandemic--Statistics and Research [Internet]. [cited 2022 Jan 26]. Available from: https://ourworldindata.org/policy-responses-covid

For peer review only

Figure 1. Fertility intentions throughout the pandemic, by gender
Restricted to those with complete survey data at all three time points

For peer review only

For peer review only - http://bmjopen.bmj.com/site/about/guidelines.xhtml

Figure 2. Contraceptive dynamics throughout the pandemic by method effectiveness, by gender
Restricted to those who were sexually active and in need of contraception (not pregnant and did not want a child soon/now or within the next year) at both the time of that survey and the prior survey round, per gender; Method effectiveness: LARC=IUD, implant; short-acting=injectable, pills; coital-dependent=emergency contraception, male condoms, female condoms; other/traditional=rhythm, withdrawal, herbal pill method

For peer review only

Figure 3. Main reason for discontinuation at 12-month (n=143) and 18-month (n=137) follow-up

Pregnancy-related: want to become pregnant; Partner-related: fear of cheating, partner opposed, partner away, infrequent sex, not married; COVID-related: could not go to facility due to COVID-19 restrictions; Access-related: lack of access/too far, cost, preferred method not available, no method available, inconvenient to use, knows no alternate method; Personal concerns about/opposition to contraception: respondent opposed, interferes with body's processes, religious reasons, others opposed, fear of side effects, health concerns

For peer review only

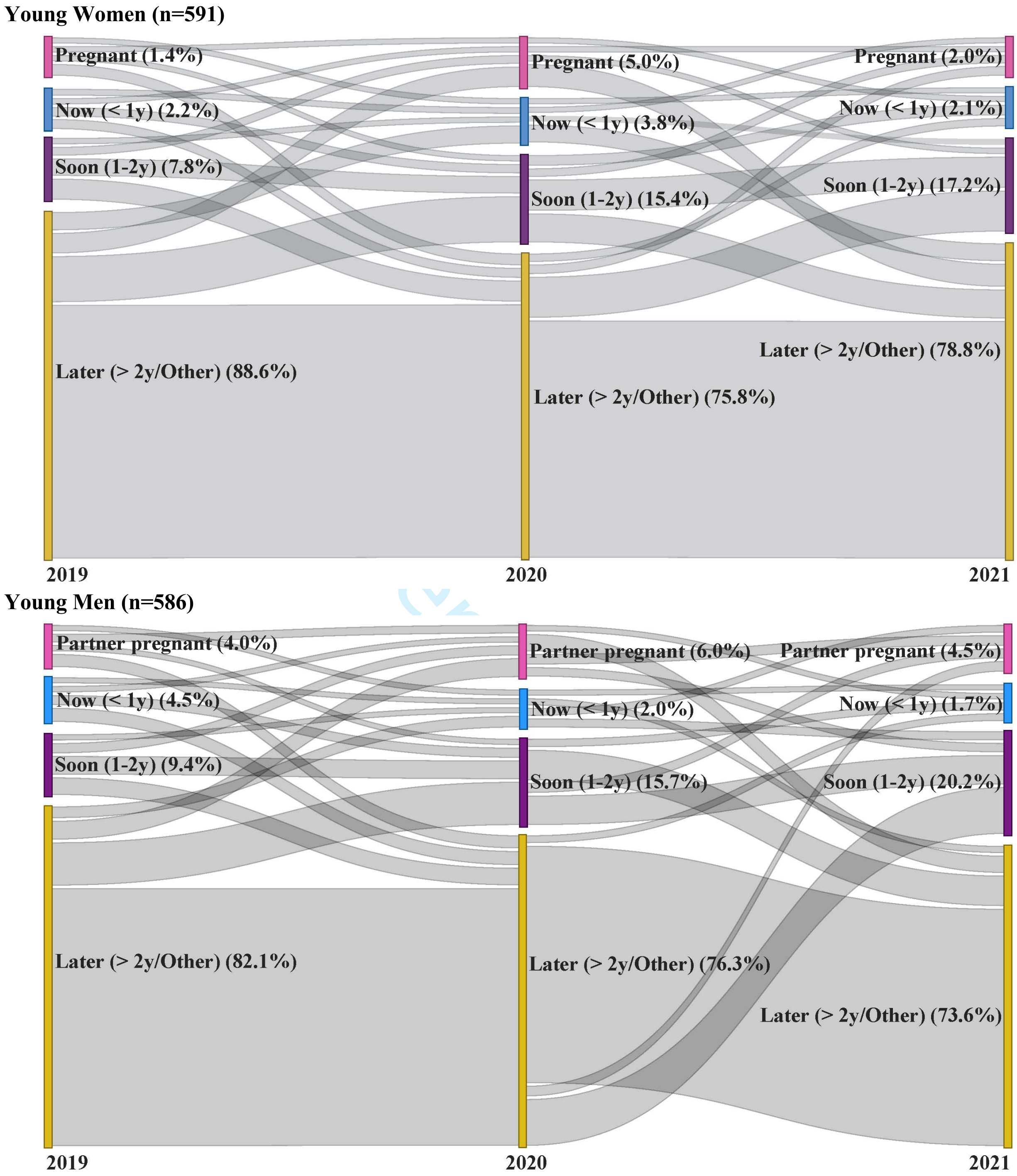
Young Women (n=591)
Pregnant (1.4%)
Now (< 1y) (2.2%)
Soon (1-2y) (7.8%)
Later (> 2y/Other) (88.6%)
Pregnant (5.0%)
Now (< 1y) (3.8%)
Soon (1-2y) (15.4%)
Later (> 2y/Other) (75.8%)
Pregnant (2.0%)
Now (< 1y) (2.1%)
Soon (1-2y) (17.2%)
Later (> 2y/Other) (78.8%)
2019
2020
2021
Young Men (n=586)
Partner pregnant (4.0%)
Now (< 1y) (4.5%)
Soon (1-2y) (9.4%)
Later (> 2y/Other) (82.1%)
Partner pregnant (6.0%)
Now (< 1y) (2.0%)
Soon (1-2y) (15.7%)
Later (> 2y/Other) (76.3%)
Partner pregnant (4.5%)
Now (< 1y) (1.7%)
Soon (1-2y) (20.2%)
Later (> 2y/Other) (73.6%)
2019
2020
2021

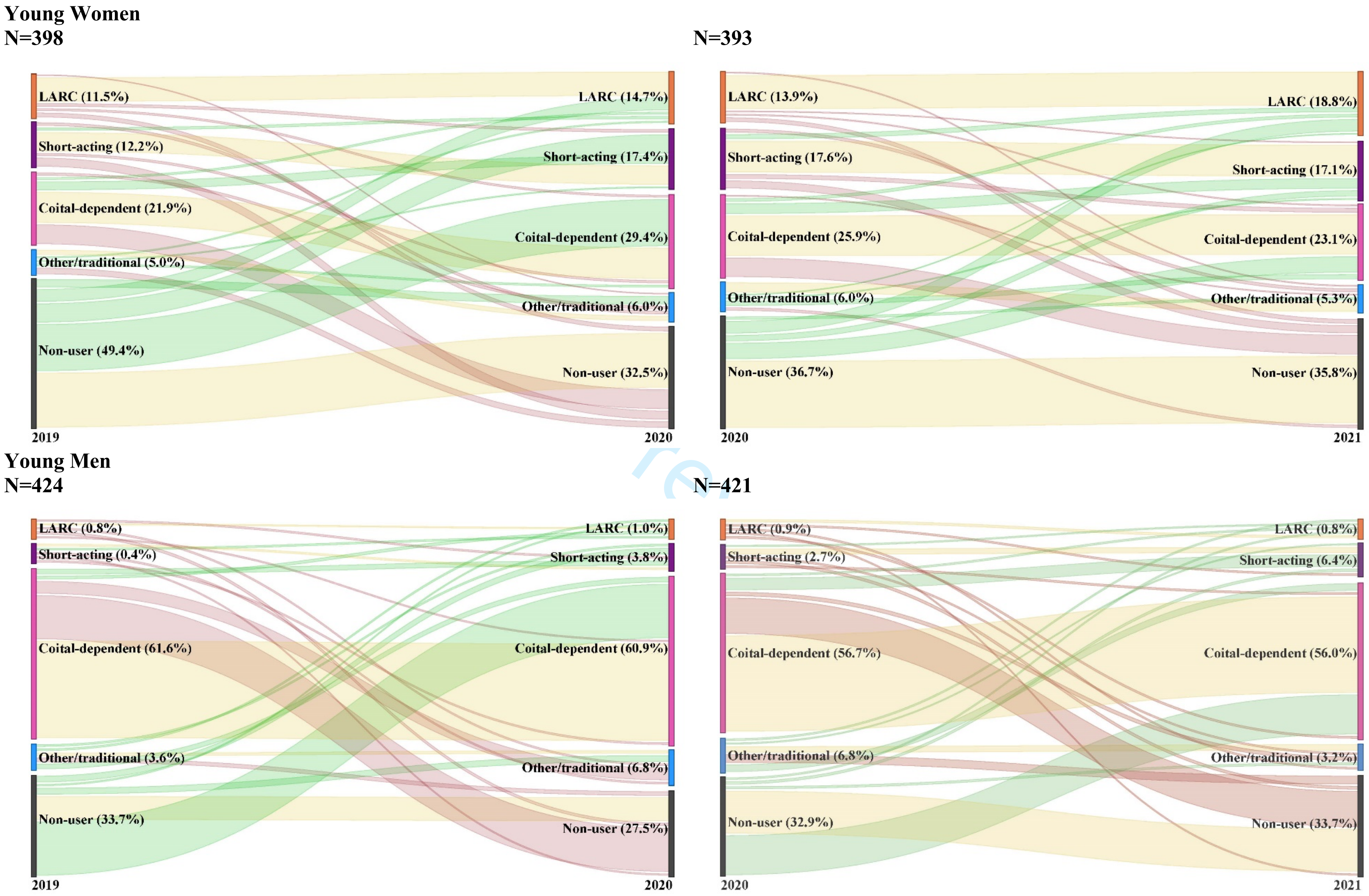
Young Women
N=398
LARC (11.5%)
Short-acting (12.2%)
Coital-dependent (21.9%)
Other/traditional (5.0%)
Non-user (49.4%)
LARC (14.7%)
Short-acting (17.4%)
Coital-dependent (29.4%)
Other/traditional (6.0%)
Non-user (32.5%)
2019
2020
N=393
LARC (13.9%)
Short-acting (17.6%)
Coital-dependent (25.9%)
Other/traditional (6.0%)
Non-user (36.7%)
LARC (18.8%)
Short-acting (17.1%)
Coital-dependent (23.1%)
Other/traditional (5.3%)
Non-user (35.8%)
2020
2021
Young Men
N=424
LARC (0.8%)
Short-acting (0.4%)
Coital-dependent (61.6%)
Other/traditional (3.6%)
Non-user (33.7%)
LARC (1.0%)
Short-acting (3.8%)
Coital-dependent (60.9%)
Other/traditional (6.8%)
Non-user (27.5%)
2019
2020
N=421
LARC (0.9%)
Short-acting (2.7%)
Coital-dependent (56.7%)
Other/traditional (6.8%)
Non-user (32.9%)
LARC (0.8%)
Short-acting (6.4%)
Coital-dependent (56.0%)
Other/traditional (3.2%)
Non-user (33.7%)
2020
2021

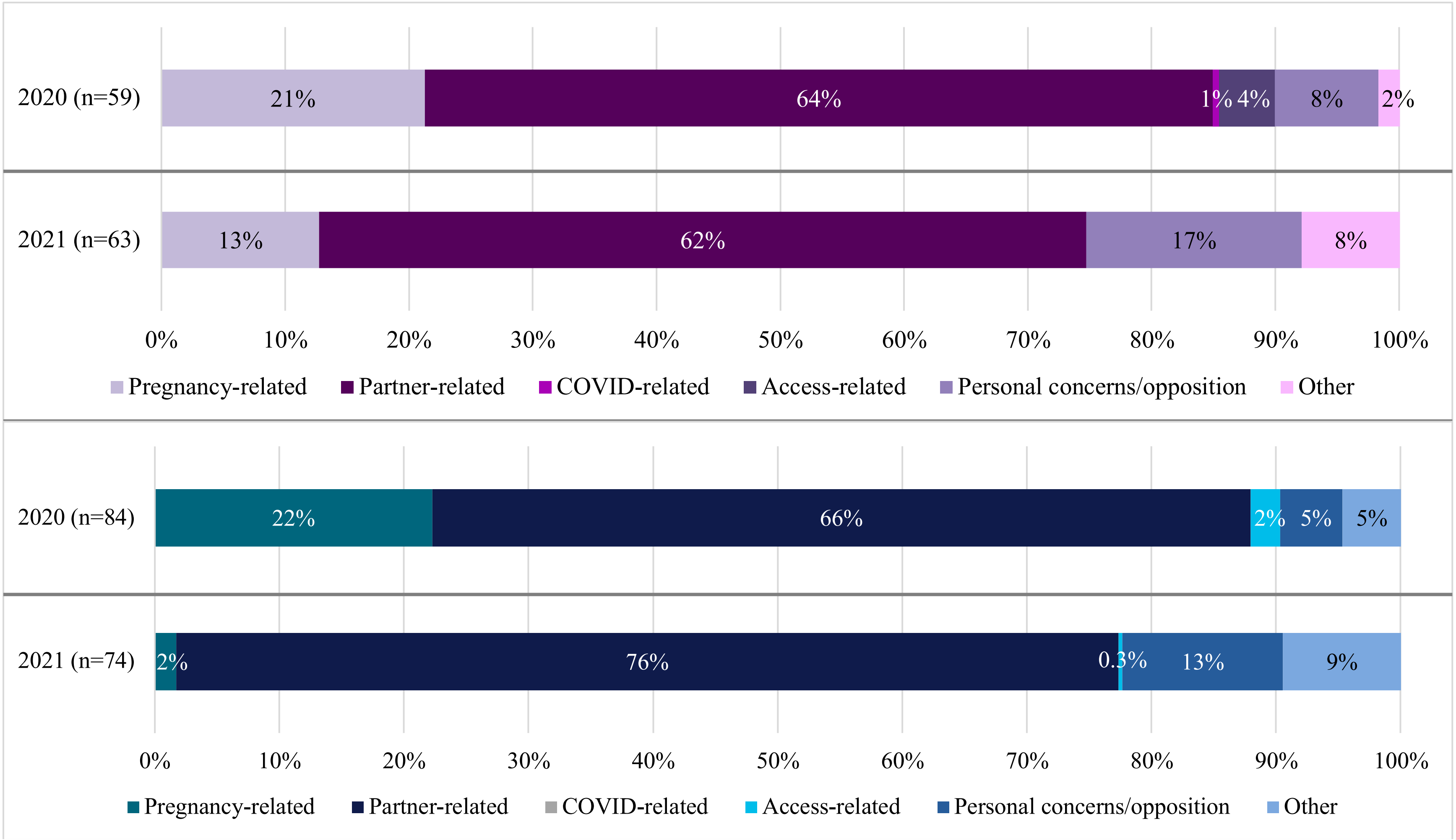
2020 (n=59)
21%
64%
1%
4%
8%
2%
2021 (n=63)
13%
62%
17%
8%
0% 10% 20% 30% 40% 50% 60% 70% 80% 90% 100%
Pregnancy-related
Partner-related
COVID-related
Access-related
Personal concerns/opposition
Other
2020 (n=84)
22%
66%
2%
5%
5%
2021 (n=74)
2%
76%
0.3%
13%
9%
0% 10% 20% 30% 40% 50% 60% 70% 80% 90% 100%
Pregnancy-related
Partner-related
COVID-related
Access-related
Personal concerns/opposition
Other

STROBE Statement—Checklist of items that should be included in reports of ***cohort studies***

	Item No	Recommendation	Page No
**Title and abstract**	1	(*a*) Indicate the study's design with a commonly used term in the title or the abstract	1
		(*b*) Provide in the abstract an informative and balanced summary of what was done and what was found	2
**Introduction**			
Background/rationale	2	Explain the scientific background and rationale for the investigation being reported	4
Objectives	3	State specific objectives, including any prespecified hypotheses	4
**Methods**			
Study design	4	Present key elements of study design early in the paper	5
Setting	5	Describe the setting, locations, and relevant dates, including periods of recruitment, exposure, follow-up, and data collection	5
Participants	6	(*a*) Give the eligibility criteria, and the sources and methods of selection of participants. Describe methods of follow-up	5
		(*b*) For matched studies, give matching criteria and number of exposed and unexposed	NA
Variables	7	Clearly define all outcomes, exposures, predictors, potential confounders, and effect modifiers. Give diagnostic criteria, if applicable	5
Data sources/ measurement	8*	For each variable of interest, give sources of data and details of methods of assessment (measurement). Describe comparability of assessment methods if there is more than one group	5
Bias	9	Describe any efforts to address potential sources of bias	8
Study size	10	Explain how the study size was arrived at	6
Quantitative variables	11	Explain how quantitative variables were handled in the analyses. If applicable, describe which groupings were chosen and why	5
Statistical methods	12	(*a*) Describe all statistical methods, including those used to control for confounding	6
		(*b*) Describe any methods used to examine subgroups and interactions	6
		(*c*) Explain how missing data were addressed	6
		(*d*) If applicable, explain how loss to follow-up was addressed	6
		(*e*) Describe any sensitivity analyses	NA
**Results**			
Participants	13*	(a) Report numbers of individuals at each stage of study—eg numbers potentially eligible, examined for eligibility, confirmed eligible, included in the study, completing follow-up, and analysed	6
		(b) Give reasons for non-participation at each stage	6
		(c) Consider use of a flow diagram	NA
Descriptive data	14*	(a) Give characteristics of study participants (eg demographic, clinical, social) and information on exposures and potential confounders	6
		(b) Indicate number of participants with missing data for each variable of interest	6
		(c) Summarise follow-up time (eg, average and total amount)	5
Outcome data	15*	Report numbers of outcome events or summary measures over time	6

For peer review only

Main results	16	(*a*) Give unadjusted estimates and, if applicable, confounder-adjusted estimates and their precision (eg, 95% confidence interval). Make clear which confounders were adjusted for and why they were included	7
		(*b*) Report category boundaries when continuous variables were categorized	NA
		(*c*) If relevant, consider translating estimates of relative risk into absolute risk for a meaningful time period	NA
Other analyses	17	Report other analyses done—eg analyses of subgroups and interactions, and sensitivity analyses	7
**Discussion**			
Key results	18	Summarise key results with reference to study objectives	8
Limitations	19	Discuss limitations of the study, taking into account sources of potential bias or imprecision. Discuss both direction and magnitude of any potential bias	8
Interpretation	20	Give a cautious overall interpretation of results considering objectives, limitations, multiplicity of analyses, results from similar studies, and other relevant evidence	8
Generalisability	21	Discuss the generalisability (external validity) of the study results	8
**Other information**			
Funding	22	Give the source of funding and the role of the funders for the present study and, if applicable, for the original study on which the present article is based	10

*Give information separately for exposed and unexposed groups.

Note: An Explanation and Elaboration article discusses each checklist item and gives methodological background and published examples of transparent reporting. The STROBE checklist is best used in conjunction with this article (freely available on the Web sites of PLoS Medicine at http://www.plosmedicine.org/, Annals of Internal Medicine at http://www.annals.org/, and Epidemiology at http://www.epidem.com/). Information on the STROBE Initiative is available at http://www.strobe-statement.org.

For peer review only

For peer review only - http://bmjopen.bmj.com/site/about/guidelines.xhtml
